# ATGL suppresses ferroptosis in acute myeloid leukemia cells by modulating the CEBPα/SCD1 axis and induces gilteritinib resistance

**DOI:** 10.1038/s41419-025-08388-0

**Published:** 2026-01-09

**Authors:** Shiyi Yuan, Ying Zhou, Wenrui Xiao, Ning Liu, Ping Zhang, Ying Zhang, Jianchuan Deng, Liang Fang, Xi Zhang, Shifeng Lou

**Affiliations:** 1 Department of Hematology, The Second Affiliated Hospital of Chongqing Medical University, Chongqing Medical University, Chongqing, China; 2 People’s Hospital of Qianxinan Prefecture, Xingyi, Guizhou Province China; 3 Growth, Development and Mental Health Center of Children and Adolescents, National Clinical Research Center for Child Health and Disorders, Children’s Hospital of Chongqing Medical University, Chongqing Medical University, Chongqing, China; 4 Medical Center of Hematology, Xinqiao Hospital, Army Medical University, Chongqing, Chongqing, China; 5 State Key Laboratory of Trauma and Chemical Poisoning, Chongqing Key Clinical Specialty, Chongqing Key Laboratory of Hematology and Microenvironment, Chongqing, Chongqing, China; 6 Jinfeng Laboratory, Chongqing, Chongqing, China

**Keywords:** Acute myeloid leukaemia, Cancer metabolism

## Abstract

Metabolic reprogramming disrupts energy homeostasis and promotes tumor cell proliferation. In the present study, high expression of adipose triglyceride lipase (ATGL) in patients with acute myeloid leukemia (AML) predicted a poor clinical prognosis. Furthermore, the aberrant upregulation of ATGL was confirmed to promote the malignant progression of AML through gene ablation, overexpression, and pharmacological inhibition of ATGL, particularly in FLT3-ITD-mutated AML. RNA sequencing, lipid peroxidation, cellular iron, and ROS assays were performed to confirm the association of ATGL with ferroptosis. Mechanistically, ATGL is positively correlated with stearoyl-CoA decarboxylase 1 (SCD1) and promotes the malignant progression of AML by inhibiting ferroptosis through the CEBPα/SCD1 axis. We established gilteritinib-resistant MOLM-13 and MV4-11 cell lines and collected cells from patients with *FLT3*-ITD mutations to confirm that ATGL inhibitors increased the efficacy of gilteritinib. Consequently, we constructed an AML xenograft model using cells derived from patients with *FLT3*-ITD-mutated AML to confirm the efficacy of combining ATGL inhibitors with gilteritinib in vivo. This study provides novel therapeutic targets and monitoring indicators for AML, along with new treatment strategies for patients with *FLT3*-ITD-mutated AML and those with relapsed/refractory *FLT3*-ITD-mutated AML.

## Introduction

Acute myeloid leukemia (AML), the most common and prevalent type of hematological malignancy, has a 5-year overall survival rate of <30% [1]. AML is characterized by the presence of malignant proliferative clones with multiple mutations in *NPM1, TP53, FLT3, KIT, RUNX1*, and other genes [2, 3]. Owing to the complexity of gene mutations, combination chemotherapy has been used for the treatment of AML. However, repeated rounds of chemotherapy can lead to drug resistance [4]. *FLT3-*mutation rates of up to 30% have been observed in patients with AML. *FLT3* gene mutations are common in patients with relapsed/refractory AML and drug-resistant, and are associated with poor treatment outcomes [2]. Gilteritinib has been widely used to treat AML with *FLT3* mutations as well as relapsed and refractory AML with *FLT3* mutations [5].

Alterations in metabolic reprogramming were considered to sustain the growth and proliferation of tumor cells by Hanahan, Weinber, and others. Metabolic reprogramming is a novel field of research in the context of the development and mechanisms of tumorigenesis [6, 7]. Recent research on AML suggests that AML cells can withstand environmental stress within the tumor microenvironment and treatment through synergistic changes in lipid metabolism, with fatty acid regulation playing a particularly crucial role in AML [8]. Abnormal lipid metabolism may contribute to treatment resistance in patients with AML, and targeting lipid metabolism in AML could address treatment resistance and clarify the metabolic context. Other researchers have suggested that leukemia stem cells evade chemotherapy by metabolically adapting to the adipose tissue microenvironment, and the abnormal expression of *ATGL* is potentially involved in this process [9]. Our previous studies have revealed that ATGL participates in the regulation of lipid metabolism in AML [10]. However, neither the specific mechanisms nor the potential roles of ATGL in AML have been revealed. Furthermore, ATGL significantly affects lipid metabolic reprogramming in other solid tumors because of its differential expression patterns.

SCD1 has been identified as a key enzyme that regulates fatty acid anabolism in other tumors. Furthermore, it is associated with ferroptosis, tumor prognosis, and drug resistance and promotes tumor progression, invasion, and metastasis in vivo [11–13]. Mechanistic studies have revealed that the abnormal transcription and epigenetic activation of SCD1 modulate AMPK/ACC, SIRT1/PGC1, and NcDase/Wnt signaling pathways and are associated with transcription factor CEBPα, thereby driving cancer progression [14]. Studies have indicated that, in response to tyrosine kinase inhibitors and chemotherapy, SCD1 is critical for tumor recurrence in the tumor microenvironment while also protecting cancer cells from oxidative stress-induced ferroptosis. This role has been validated in the formation and metastasis of malignant ascites in patients with ovarian cancer [15]. The aberrant activity of lipid metabolism leading to increased fatty acid synthesis indicates the proliferative capacity of tumor cells [16, 17]. These changes play a critical role in maintaining the aggressive nature of many malignant tumors [18]. This study aimed to show for the first time that ATGL regulates the levels of monosaturated/polyunsaturated fatty acids (MUPAs/PUPAs) in AML by modulating the SCD1, which promotes cell proliferation, inhibits apoptosis and lipid peroxide metabolism, and promotes the malignancy of AML. Furthermore, this study aimed to demonstrate the close relationship between ATGL and targeted resistance in *FLT3*-ITD-mutated AML. The combination of ATGL inhibitors with FLT3 inhibitors with or without ferroptosis inducers increases antitumor efficacy against *FLT3*-ITD-mutated AML, thereby providing a novel therapeutic strategy for patients with *FLT3*-ITD-mutated AML.

## Materials and methods

### AML-patients samples, cell lines, and culture

PBMCs and bone marrows were obtained from healthy volunteers and patients with *FLT3*-ITD-mutated AML and relapsed/refractory *FLT3*-ITD-mutated after informed consent on protocols approved by the Ethics in Research Committee of Chongqing Medical University in Chongqing, China (2021129). The HL60, THP-1, KG1-a, MOLM-13, and MV4-11 (acute leukemia cell lines), K562 (leukemia cell line), Jurkat (acute T cell leukemia cell lines), CCRF-CEM (acute T cell lymphoblastic leukemia cell line), cells were cultured in RPMI-1640 medium with 10% fetal bovine serum, penicillin/streptomycin and additional 0.05 mM beta-mercaptoethanol was added to the medium for THP-1 cells. Cells were maintained in a humidified atmosphere containing 5% carbon dioxide (CO_2_) at 37 °C. All cell lines have been routinely tested for Mycoplasma.

### Primary AML blast cells

Mononuclear cells were extracted from samples according to the Ficoll-Hypaque density centrifugation method. After extraction, erythrocytes were lysed and mixed, centrifuged again, washed with PBS, and cultured in 1640 medium plus 20% FBS, ITS Solution (Sigma-Aldrich, St Louis, MO, USA), and 20% supernatant of the 5637 bladder cancer cell line [19, 20].

### Virus infection

Lentiviral vectors for *ATGL* overexpression (NM_001113291.2), *SCD1* (NM_005063.5), and *CEBPα* (NC_000019.10) were purchased from Shenggong (Shanghai, China), and an empty vector was used as the negative control (NC). Green fluorescent protein (GFP) was used to assess the transduction efficiency, and puromycin was used to select stably transduced cells. The shRNA sequences targeting *ATGL* were designed and synthesized by Tsingke Company (Beijing, China). The relevant sequences are listed in Supplementary Table S1. All transfections were performed according to the manufacturer’s instructions.

### RNA extraction and real-time quantitative-PCR analysis

Total RNA was extracted from cells using TRIzol reagent (Invitrogen, Carlsbad, CA, USA). RNA was synthesized into cDNA using the SuperScript™ VILO™ cDNA Synthesis Kit (Thermo Fisher). Reverse transcription-quantitative PCR (RT-qPCR) was performed using SYBR Premix Ex TaqTM (Tli RNaseH Plus) (TaKaRa, Dalian, China). Expression was normalized to the housekeeping control GAPDH. The amplification primers are shown in Table S1.

### Cell proliferation, apoptosis, and cell cycle assays

Cell proliferation was assessed using Cell Counting Kit-8 (G4000, Promega, Madison, WI). The apoptosis rate was assessed using an Annexin V-FITC/PI apoptosis detection kit (Elabscience Biotechnology Co., Ltd., Wuhan, China), and the cell cycle was assessed using a Cell Cycle Analysis Kit (Sigma, St. Louis, MO, USA) by flow cytometry (FCM).

### MUFAs and PUFAs

PUFAs concentration was assessed by the PUFAs Elisa Kit (JingMei Biotechnology, Jiangsu, China) according to the manufacturer’s instructions. MUFAs concentration was assessed by MUFAs Elisa Kit (JingMei Biotechnology, Jiangsu, China) according to the manufacturer’s instructions.

### Cellular ATP level measurement

ATP levels were measured by the ATP Assay Kit (S0026, Beyotime), 2 mL of indicated cells (containing 2 × 10^5^ cells) was transferred into a luminometer plate.

### Synegistic effect in vitro cell assay

Approximately 5000 cells were cultured in each well of a 96-well plate and cultured under conditions of 37 °C, 5% CO_2_, and 95% humidity. They were treated with different concentrations of Atglistatin, Gilteritinib, and Erastin, in Combination drug, with a blank control group included. After 24 h of treatment, cell absorbance (OD) was measured using the CCK8 assay kit, with data read at 450 nm wavelength via a multimode reader (Molecular Devices, USA). Cell survival rates at different drug concentrations were calculated using the formula to determine the IC_50_ values. Drug synergistic effects were assessed using the combination index (CI) formula based on the Chou–Talalay multiple drug effect equation. The combination index (CI) was calculated using CompuSyn software (Combosyn Inc., Paramus, NJ, USA) to determine the degree and direction of synergistic antileukemic effects between the two or three drugs. CI < 1 indicates synergistic effects, CI = 1 indicates additive effects, and CI > 1 indicates antagonistic effects [21, 22].

### RNA-seq and data analysis

The RNA samples were prepared well and sent to Baiqu Technology (Shanghai, China). Detailed methodology is available in the Supplementary Information; the data have been uploaded to the GEO database GSE307476.

### ROS assay, iron assay

Use the OxiSelect™ intracellular ROS assay kit to analyze ROS after different treatments by FCM, according to the instructions. The iron standard was prepared, and the cell lysate was homogenized and diluted, and tested by FCM following the Iron Assay Kit (Scien Cell) protocol.

### Lipid peroxidation

To evaluate lipid peroxidation in MOLM-13 and MV4-11 cells, the cells were treated with drug treatments for 24 h. Cells were stained with fluorescent dye C11-BODIPY581/591 (Thermo Fisher) at 37 °C with a probe (2.0 µM) for 30 min, Lipid peroxidation was detected using an inverted microscope and analyzed by a BD LSRII FCM (Becton Dickinson). The cells were detached and washed with PBS before FITC fluorescence intensity (oxidized BODIPY emission) was measured using a CytoFLEX (Beckman Coulter) flow cytometer. FlowJo software was used to assess the geometric mean of the intensity of oxidized BODIPY 581/591 C11.

### Western blot

Cells were lysed with RIPA lysis buffer. Protein was separated by SDS–PAGE and transferred to PVDF membranes. The membranes were blocked with 5% non-fat milk, incubated sequentially with primary and secondary antibodies, and detected by immunoblotting with the Pierce ECL Western Blotting Substrate. Immunoblot imaging was performed using the ChemiDoc MP Imaging System (Bio-Rad, USA), anti-ATGL (DF7277, Affinity,1:1000), anti-SCD1 (DF13253, Affinity, 1:1000), anti-GAPDH (HRP-60004, Proteintech, 1:1000), anti-Cleave-Caspase3 (BF0711, Affinity, 1:1000), anti-Bax (AF0120, Affinity, 1:1000), anti-Bcl-2 (AF6139, Affinity, 1:1000), and anti-CEBPα (AF6333, Affinity, 1:500).

### Hematoxylin–eosin staining (HE)

The sections of mouse spleen and liver were embedded and dewaxed for 30 min, incubated in a high-power microwave oven for 8 min, hematoxylin reverse staining for 8 min, and blue dye reverse staining for 4 min. Slide into eosin staining solution and stain for 1–3 min. The slides are then dehydrated and transparent, the sections are taken out of the xylene and slightly dried, sealed with a neutral glue, and the sample is then evaluated using a microscope.

### Immunohistochemistry (IHC) analysis

The sections were treated with 3% hydrogen peroxide and methanol for 30 min at room temperature. The samples were blocked with goat serum and incubated with a primary antibody at 4 °C overnight. The samples were treated with a secondary antibody, and 3,3-diaminobenzidine (DAB) was used as a chromogenic agent to evaluate the samples using a microscope. Finally, hematoxylin reagent was added to stain the nuclei, and the samples were sealed using neutral gum. The following antibodies were used: anti-ATGL (DF7277, Affinity,1:100) and anti-SCD1 (DF13253, Affinity, 1:100).

### AML xenograft model

All animal experiments were performed according to protocols approved by the Animal Ethics Committee of Chongqing Medical University (Approval No. 2021129) and adhered to the 3Rs principle. 4–5 weeks old NOD.Cg-PrkdcscidIl2rgtm1Wjl/SzJ (NSG) mice (11–12 g) were purchased from the Vital River (Beijing, China). For in vivo experiments, they were injected via the tail vein with 5 × 10^6^ MV4-11 cells (in 100 μl PBS). To minimize bias, mice were randomly assigned to experimental groups using a computer-generated randomization protocol. Randomization was performed after acclimatization, ensuring equal distribution based on body weight to maintain homogeneity between groups. One week after injection of leukemic cells, animals were randomized into 3 groups: ShNC, Sh*ATGL*-1, Sh*ATGL*-2 (3 groups, *n* = 4). Another set of models was used to look at survival time (3 groups, *n* = 5). In another experiment, Animals were randomized into 2 groups: NC, *ATGL*-OE (3 groups, *n* = 4), and another set of models was used to look at survival time (3 groups, *n* = 5). In patient-derived xenograft (PDX) experiments, we collected bone marrow from patients with R/R *FLT3*-ITD-mutated AML and extracted single-nucleated cells (Table S2). Animals were randomized into the following treatment groups (7 groups, *n* = 3): Vehicle (25% DMSO, 50% phosphate-buffered saline), ATGL-inhibitor (Atglsitation 30 mg/kg, po, once daily), Gilteritinib (10 mg/kg, po, once daily), Atglsitation + Gilteritinib, Erastin (15 mg/kg, iv, once daily), Atglsitation + Erastin, Atglsitation + Gilteritinib + Erastin. For assessment of leukemia burden, the mice were euthanized after the end of drug treatment. Harvested bone marrow cells were analyzed for human CD45 expression by FCM, and compared the size of spleen and liver, compared the mice weight. Spleen and liver were performed by HE and ICH. For survival analysis, treatment was initiated on day 7 after transplantation and continued until day 35. Moribund animals were euthanized when they displayed signs of terminal leukemic disease; the final observation time was 120 days.

### Quantification and statistical analysis

Data were analyzed and presented as mean ± SD. A two-tailed Student’s *t*-test was used to compare means between groups as indicated; *p* < 0.05 was considered significant. All experiments were performed in triplicate unless otherwise noted. Graph Pad Prism version 9.0 was used for all statistical tests and plot generation.

## Results

### Transcriptome sequencing and analysis of the TCGA database reveal ATGL as a key regulator of lipid metabolic reprogramming in AML

**Fig. 1:**
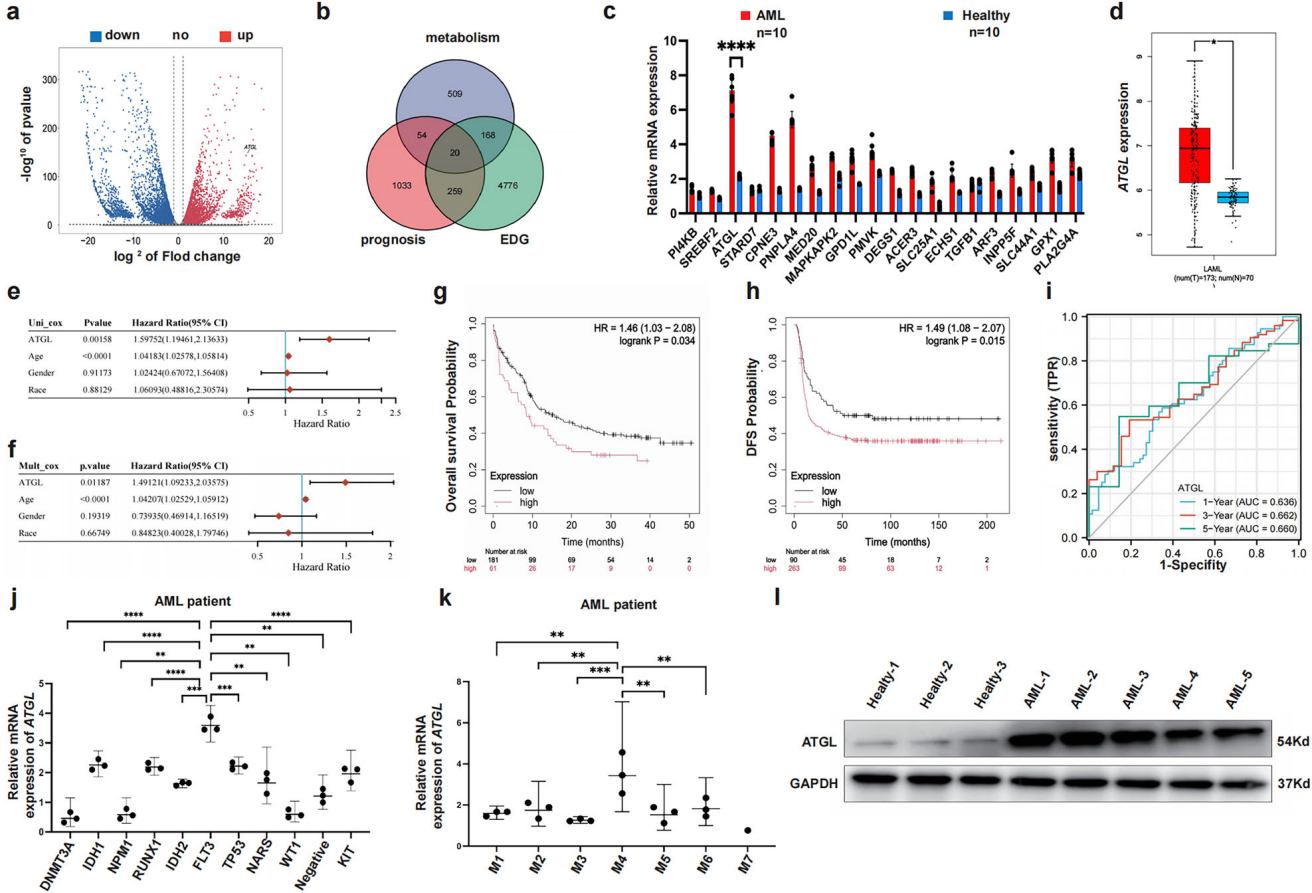
Transcriptome sequencing and TCGA database analysis reveal ATGL as a key regulator of lipid metabolism in AML. **a** Volcano map showing gene expression from transcriptome sequencing of healthy individuals and patients with AML. **b** Venn diagram showing lipid metabolism-related genes screened by the TCGA database. **c** RNA expression of 20 genes related to lipid metabolism in AML by RT-qRCR. **d** Comparison of ATGL expression in healthy individuals and patients with AML using the TCGA database. **e** and **f** Unifactorial and multifactorial analysis of ATGL in AML. **g–i** Kaplan–Meier survival analysis showing overall survival, disease-free survival, and survival time ROC based on TCGA dataset analysis in patients with AML with high expression of ATGL. **j** The RNA expression of ATGL in different gene mutations of AML. **k** RNA expression of ATGL in different types of AML. **l** Protein levels of ATGL in healthy individuals and patients with AML by Western blot. *P < 0.05; **P < 0.01; ***P < 0.001; ns not significant.

A comparison between the transcriptome sequencing data from patients with AML and those from healthy individuals in a previous study revealed that ATGL was abnormally upregulated in patients with AML (Fig. 1a). RT-qPCR conducted using 20 target genes related to lipid metabolism selected from the TCGA database revealed a significant increase in the expression of *ATGL* mRNA in patients with AML (Fig. 1b and c). A comparison of *ATGL* expression between patients with AML and healthy individuals in the TCGA database also indicated that *ATGL* expression was upregulated in patients with AML (Fig. 1d). Multifactorial univariate analyses revealed associations between elevated levels of ATGL and various other factors in patients with AML (Fig. 1e and f). The overall survival, survival curves, and disease-free survival rates indicated that the abnormal upregulation of *ATGL* expression was associated with decreased patient survival and a poor prognosis (Fig. 1g–i). We subsequently validated *ATGL* expression in patients with AML with different gene mutations and in patients with different AML subtypes. These results suggested that *ATGL* expression was most strongly associated with *FLT3* gene mutations and the M4 subtype (Fig. 1j and k). Next, Western blotting was performed to compare the levels of the ATGL protein in the bone marrow of patients with AML and healthy individuals. The results revealed its upregulation in patients with AML (Fig. 1l). We collected clinical data and treatment information from patients with 119 leukemia, who were allocated to two groups based on high and low *ATGL* expression, to better evaluate the clinical significance of ATGL (Tables 1 and 2). The results revealed that the chemotherapy remission rate was reduced, the disease recurrence rate was increased, and the survival rate was reduced among the patients in the high ATGL group.

**Table 1:** Comparison of clinical and molecular characteristics with the ATGL expression status in patients with AML.

Characteristic	Total	ATGL-low (*n* = 50)	ATGL-high (*n* = 69)	*P* value
**Age/years, median (range)**		61.5 (22–82)	63 (15–81)	0.936*
**Age group/*n*** **(%)**				0.740§
<60 years	55	24 (48%)	31 (44.9%)	
≥60 years	64	26 (52%)	38 (55.1%)	
**Sex/*n*** **(%)**				0.329§
Male	70	32 (64%)	38 (55.1%)	
Female	49	18 (36%)	31 (44.9%)	
**WBC×10**^**9**^**/L, median (range)**		8.09 (1–152)	4.68 (0–411)	0.181*
**BM blasts/%, median (range)**		41 (2–98)	47 (1–94)	0.338*
**FAB subtype/*n*** **(%)**				
M0	9	4 (8%)	5 (7%)	0.739§
M1	12	4 (8%)	8 (12%)	0.248§
M2	41	22 (44%)	19 (28%)	0.639§
M3	6	2 (4%)	4 (6%)	0.414§
M4	15	7 (14%)	8 (12%)	0.796§
M5	4	1 (2%)	3 (4%)	0.317§
M6	3	3 (6%)	0 (0%)	**0.000§**
MDS	26	7 (14%)	19 (28%)	**0.019§**
Other (CMML/CML)	3	2 (4%)	1 (1%)	0.564§
**Cytogenetics/*n*** **(%)**				
Normal	55	30 (60%)	22 (32%)	0.267§
Complex karyotype	18	3 (6%)	23 (33%)	**0.000§**
Other	46	17 (34%)	24 (35%)	0.274§
**Pretreatment assessment (%)**				
Initial treatment	89	40 (80%)	49 (71%)	0.168§
Refractory/Relapse	30	10 (27%)	20 (95%)	0.273§
**Induction state after chemotherapy (%)**				
CR	58	37 (74%)	21 (30%)	**0.036§**
PR	12	3 (6%)	9 (13%)	0.083§
NR	10	0 (0%)	10 (14%)	**0.000§**
Other (death)	39	10 (20%)	29 (42%)	**0.002§**
**Multiple rounds of chemotherapy (%)**				
CR	18	9 (18%)	9 (13%)	1.000§
Autologous/allogeneic transplantation	21	9 (18%)	12 (17%)	0.513§
Refractory	11	6 (12%)	5 (7%)	0.763§
Other (untreated/death/lost to follow-up)	69	26 (52%)	43 (62%)	**0.041§**
**Survival status/*n*** **(%)**				**0.001§**
Deceased	69	19 (38%)	48 (70%)	
Living	50	31 (62%)	21 (30%)	

Bold values identify statistical significance (*p* < 0.05).

*WBC* white blood cell, *BM* bone marrow, *FAB* French–American–British.

*Mann–Whitney *U* test, ‘§’ denotes chi-square test. “Complex karyotype” is defined as the presence of more than or equal to 3 chromosomal abnormalities.

**Table. 2:** Univariate and multivariate analysis of the relationship between ATGL expression and overall survival in patients with AML.

Variable	Univariate analysis			Multivariate analysis		
	HR	95% CI	*P* value	HR	95% CI	*P* value
Sex (male vs. female)	1.215	0.747–1.976	0.433			
BM blasts (≥90% vs. <90%)	0.596	0.257–1.382	0.228			
Induction state after chemotherapy	0.539	0.33–0.879	0.013	0.83	0.486–1.418	0.495
WBC (>50*10^9^/L vs. ≤50*10^9^/L)	0.486	0.253–0.933	0.03	0.711	0.354–1.427	0.337
Complex_Cyto (yes vs. no)	0.474	0.276–0.814	0.007	0.762	0.419–1.386	0.373
ATGL_expression (high vs. low)	0.349	0.204–0.596	0	0.422	0.233–0.767	0.005

*WBC* white blood cell, *BM* bone marrow, *HR* hazard ratio, *CI* confidence interval.

Bold values identify statistical significance (*p* < 0.05). Variables with *P* < 0.05 in the univariate analysis were included in the multivariate analysis.

Collectively, these findings indicate that the abnormal upregulation of ATGL is negatively correlated with the treatment and prognosis of AML and that this effect is most significant in patients with AML carrying a mutation in *FLT3*-ITD. Thus, the upregulation of ATGL can be used as a key indicator for assessing the treatment and prognosis of AML.

### ATGL promotes the malignant progression of AML in vitro and in vivo

**Fig. 2:**
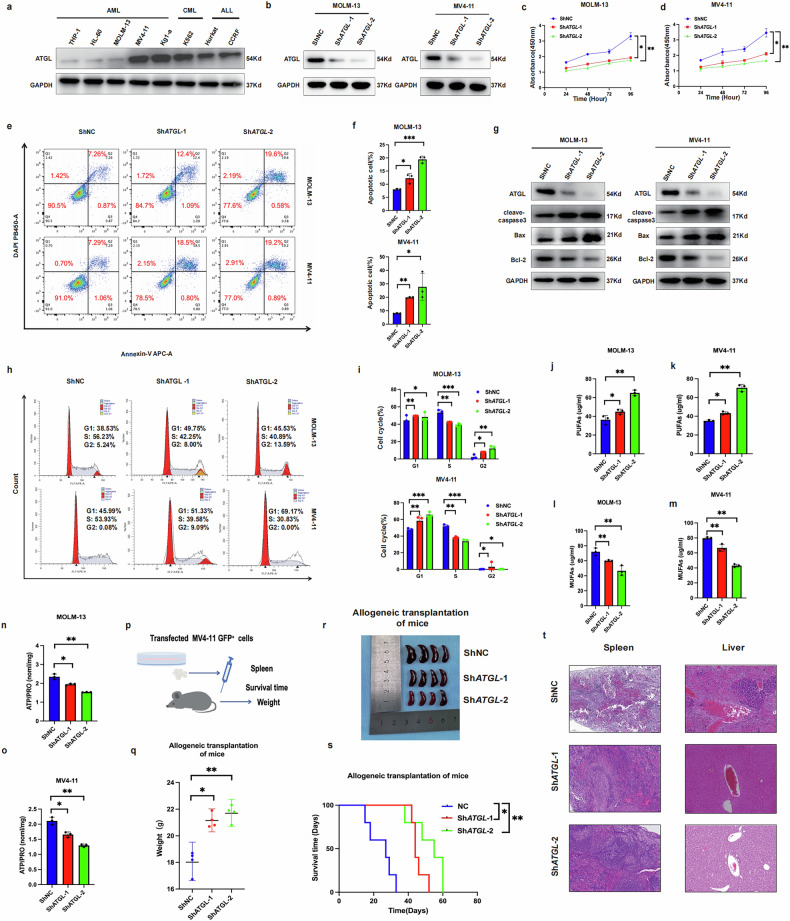
Knockdown of *ATGL* delayed the malignant progression of AML both in vivo and in vitro. **a** Determination of ATGL protein levels in hematological cell lines by Western blotting. **b** The inhibitory efficiency of ATGL shRNAs (*ATGL*-1 and *ATGL*-2) delivered by lentiviral vectors in MOLM-13 and MV4-11 cells was confirmed by Western blotting, and GAPDH was used as an internal eference. **c** and **d** Cell proliferation of MOLM13 and MV4-11 cells was measured by CCK8 assay at different time points (0, 24, 48, 72 and 96 h) after sh RNA transfection. **e** and **f** Flow cytometry (representative images are shown) was used to confirm apoptosis after *ATGL* knockdown. **g** Levels of proteins associated with apoptosis were detected by Western blotting. **h** and **i** Flow cytometry (representative images are shown) was used to analyze the cell cycle. **j–m** Changes in PUFAs and MUFAs in *ATGL*-knockdown MOLM-13 and MV4-11 cells. **n** and **o** Changes in ATP in *ATGL*-knockdown MOLM-13 and MV4-11 cells. **p** Schematic of establishing an AML allogeneic mouse model using stable MV4-11 cells after *ATGL* knockdown. **q** Final body weight of AML mice. **r** Spleen changes in *ATGL*-knockdown AML mice versus controls. **s** Survival time of AML mice with 60 days as the final observation day. **t** Histopathological staining of spleen and liver in AML mice (×40). *P < 0.05; **P < 0.01; ***P < 0.001; ns not significant.

We assessed the protein level of ATGL in leukemia cell lines through western blotting, and the results showed that the ATGL protein level was significantly higher in two cell lines, namely, the FLT3-ITD*-*mutant MOLM-13 and MV4-11 cell lines (Fig. 2a). We further elucidated the role of ATGL in AML pathogenesis by knocking down *ATGL* in MOLM-13 and MV4-11 cells and confirmed its knockdown efficiency through western blotting (Fig. 2b). *ATGL* knockdown significantly inhibited the proliferation and increased the apoptosis of MOLM-13 and MV4-11 cells (Fig. 2c–f). Western blotting analysis revealed that the levels of the antiapoptotic protein Bcl-2 were significantly decreased, whereas the levels of proapoptotic proteins (Bax and cleaved caspase-3) were slightly increased (Fig. 2g). The cell cycle analysis revealed a decrease in the proportion of AML cells in S phase after *ATGL* knockdown (Fig. 2h and i).

Next, we further examined the changes in the levels of fatty acids and ATP in MOLM-13 and MV4-11 cells with *ATGL* knockdown. Polyunsaturated fatty acid (PUFAs) levels increased, but monounsaturated fatty acid (MUFAs) and ATP levels decreased after *ATGL* knockdown (Fig. 2j–o). We used the stably transfected *ATGL-*knockdown cells MV4-11 to construct an allogeneic mouse model (3 groups, *n* = 4) to explore the effect of ATGL on AML (Fig. 2p). The mice were sacrificed 28 days after transplantation. The final body weight and changes in body weight were recorded, and the spleen size and pathological changes were compared (Figs. 2q, r and S2a). Another set of experiments (3 groups, *n* = 5) was conducted to compare the survival time of mice with allogeneic transplants, with 60 days set as the final observation period (Fig. 2t). The survival time of the Sh*ATGL*-2 group was significantly longer than that of the NC group. Next, we overexpressed *ATGL* in the MOLM-13 and MV4-11 cells, and the efficiency of ATGL protein level was confirmed through western blotting (Fig. S1a). *ATGL* overexpression promoted the proliferation and reduced the apoptosis of MOLM-13 and MV4-11 cells (Fig. S1b–g). The apoptosis analysis revealed a decrease in apoptosis in MOLM-13 and MV4-11 cells after *ATGL* overexpression (Fig. S1d–f). Western blotting showed that *ATGL* overexpression increased the expression of antiapoptotic proteins (Bcl-2) and reduced the expression of apoptotic proteins (cleaved caspase-3 and Bax) (Fig. S1g). Moreover, *ATGL* overexpression increased the percentage of MOLM-13 and MV4-11 cells in S phase (Fig. S1h–j). Then, polyunsaturated fatty acid levels decreased, but monounsaturated fatty acid and ATP levels increased after *ATGL* was overexpressed (Fig. S1k-–m). Next, we established two groups of AML mouse models with MV4-11 cells stably overexpressing *ATGL*. The body weight, spleen size, and pathological structures of the spleen and liver of AML xenograft model mice were evaluated 28 days after transplantation (2 groups, *n* = 4) (Figs. S1n–p and S2b). Another set of animal experiments conducted under the same conditions to evaluate the survival rate of mice revealed a reduction in the survival time of the mice in the *ATGL*-OE group (2 groups, *n* = 5) (Fig. S1q). Furthermore, *ATGL* expression was low in HL60 cells. We also conducted complementary experiments in HL60 cells, which showed that *ATGL* exerted the same effects in HL60 cells (Figs. S3 and S4). These results demonstrate that ATGL promotes the malignant progression of AML both in vivo and in vitro.

### Identification of the potential mechanism of *ATGL* in AML

**Fig. 3:**
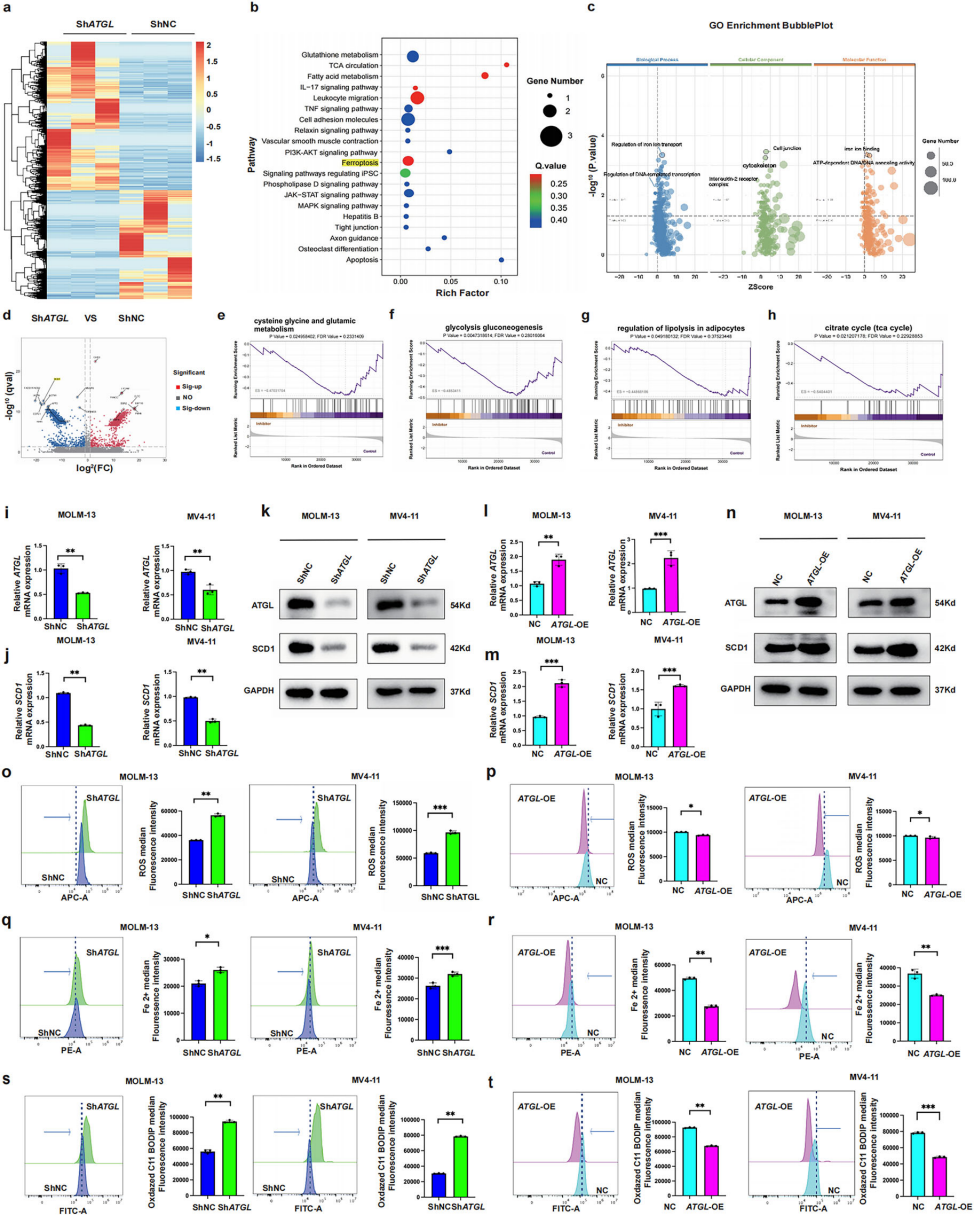
Identification of the potential ATGL targets in AML. **a** Heatmap generated from the RNA-seq data showing the representative genes after *ATGL* knockdown. **b** and **c** Kyoto Encyclopedia of Genes and Genomes (KEGG) and Gene Ontology (GO) analyses revealed the potential roles of differentially expressed genes following *ATGL* knockdown in MV4-11 cells. **d** Volcano plot of differentially expressed genes. The values on the *X* and *Y* axes in the volcano plot are the fold change (log^2^ transformed) values and *P* values (−log^10^ transformed) between the two groups, respectively. The red/blue dots indicate differentially expressed genes with a statistically significant change in expression of greater than 2-fold. **e–h** Representative GSEA results showed that fatty acid catabolism, TCA cycle, glucose metabolism, cysteine, and glutamate metabolism. **i–k** Changes in *ATGL* and *SCD1* RNA and protein levels upon *ATGL* knockdown in MOLM-13 and MV4-11 cells. **l–n** Changes in *ATGL* and *SCD1* RNA and protein levels upon *ATGL* overexpression in MOLM-13 and MV4-11 cells. **o** and **p** Changes in ROS index following *ATGL* knockdown or overexpression in MOLM-13 and MV4-11 cells. **k–r** Iron ion levels in MOLM-13 and MV4-11 cells following *ATGL* knockdown or overexpression. **s** and **t** C11-BODIPY581/591 in MOLM-13 and MV4-11 cells following *ATGL* knockdown or overexpression. *P < 0.05; **P < 0.01; ***P < 0.001; ns not significant.

We investigated the biological mechanisms by which ATGL regulates AML cells by performing RNA sequencing of *ATGL*-knockdown MV4-11 cells. Transcriptome sequencing of the *ATGL*-knockdown MV4-11 cell line, along with the control cells, revealed significant upregulation and downregulation of the expression of important genes, and the results suggested that *SCD1* is an important gene (Fig. 3a and d). KEGG analysis revealed the significant enrichment of ferroptosis and related biological functions (Fig. 3b and c). A volcano plot showed a positive correlation between *SCD1* and *ATGL* expression (Fig. 3d). GO analysis and GSEA were conducted to evaluate the role of *ATGL* and revealed that *ATGL* expression is correlated with fatty acid catabolism, the TCA cycle, glucose metabolism, and cysteine and glutamate metabolism (Fig. 3e–h).

### ATGL regulates the transcription of SCD1 in MOLM-13 and MV4-11 AML cells

We analyzed changes in ferroptosis-related genes following *ATGL* knockdown to investigate the main targets through which ATGL regulates ferroptosis in AML cells. Through a literature review [11, 13], we found that *SCD1* is a key gene that influences ferroptosis. We further validated the regulatory effects of *ATGL* on *SCD1* mRNA levels. *ATGL* knockdown significantly reduced *SCD1* mRNA levels (Fig. 3i and j), whereas *ATGL* overexpression significantly increased *SCD1* mRNA levels (Fig. 3l and m). Additionally, we examined the regulation of SCD1 protein levels by ATGL in MOLM-13 and MV4-11 cells and observed that ATGL consistently affected SCD1 protein levels in a manner corresponding to the changes in the mRNA levels (Fig. 3k and n). We subsequently performed flow cytometry to investigate changes in the levels of ROS, Fe²⁺ ions, and C11-BODIPY581/591 in MOLM-13 and MV4-11 cells with *ATGL* knockdown and overexpression. Compared with the control group, the ROS index, Fe²⁺ content, and lipid peroxidation level were significantly increased in *ATGL*-knockdown cell lines (Fig. 3o, q, and s). In contrast, *ATGL*-overexpressing cells exhibited markedly reduced ROS indices, lipid peroxidation levels, and Fe²⁺ contents (Fig. 3p, r, and t).

**Fig. 4:**
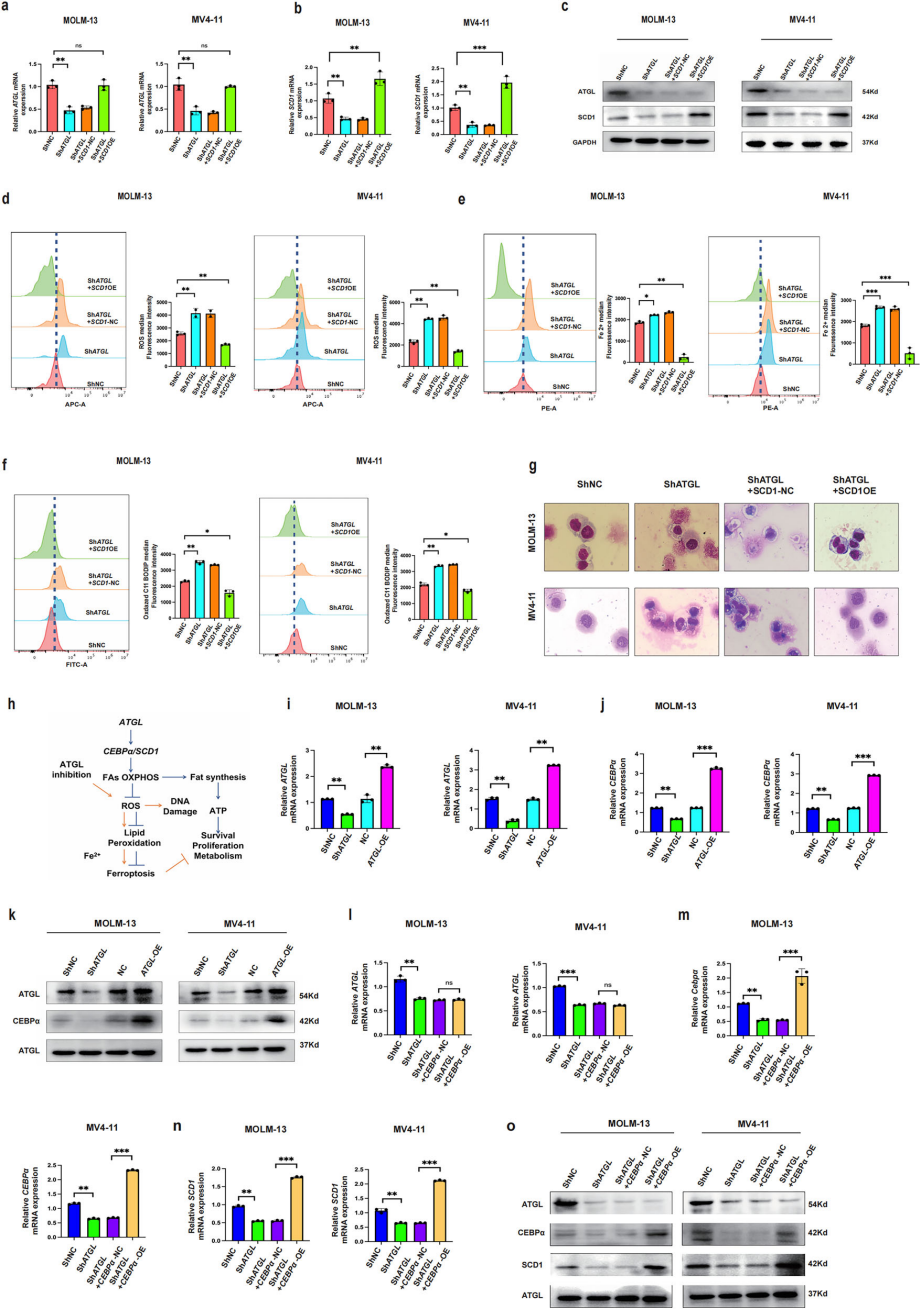
ATGL regulates ferroptosis via the CEBPα/SCD1 axis in MOLM-13 and MV4-11. **a–c** Western blotting and RT-qPCR analysis of *ATGL* and *SCD1* expression in each group. **d–f** Flow cytometry analysis of ROS index, iron ion content, and C11-BODIPY581/591 changes in each group. **g** Microscopic observation of cell changes (×40 magnification). **h** Schematic representation of the ATGL mechanism. **i–k** RNA and protein expression of ATGL and CEBPα following *ATGL* knockdown or overexpression in MOLM-13 and MV4-11. **l–o** RNA and protein expression of ATGL, SCD1, and CEBPα after *CEBPα* overexpression following *ATGL* knockdown in MOLM-13 and MV4-11.*P < 0.05; **P < 0.01; ***P < 0.001; ns not significant.

We conducted a rescue experiment to further investigate the relationship between ATGL and SCD1 regulation. *SCD1* was overexpressed in MOLM-13 and MV4-11 cells in which *ATGL* was stably knocked down. Western blot results indicated that *SCD1* overexpression failed to rescue ATGL protein levels and mRNA following ATGL knockdown (Fig. 4a–c). The flow cytometry analysis of the ROS index, Fe²⁺ content, and lipid peroxide concentration indicated that *SCD1* overexpression rescued ferroptosis in *ATGL*-knockdown MOLM-13 and MV4-11 cells (Fig. 4d–f). Direct morphological changes (×40) in these cells were observed using Wright’s staining. The images indicate that *SCD1* overexpression reversed ferroptosis-induced damage in *ATGL*-knockdown MOLM-13 and MV4-11 cells (Fig. 4g). These results indicate that ATGL regulates SCD1 and ferroptosis in AML.

### ATGL inhibits ferroptosis in AML cells via the CEBPα/SCD1 axis

**Fig. 5:**
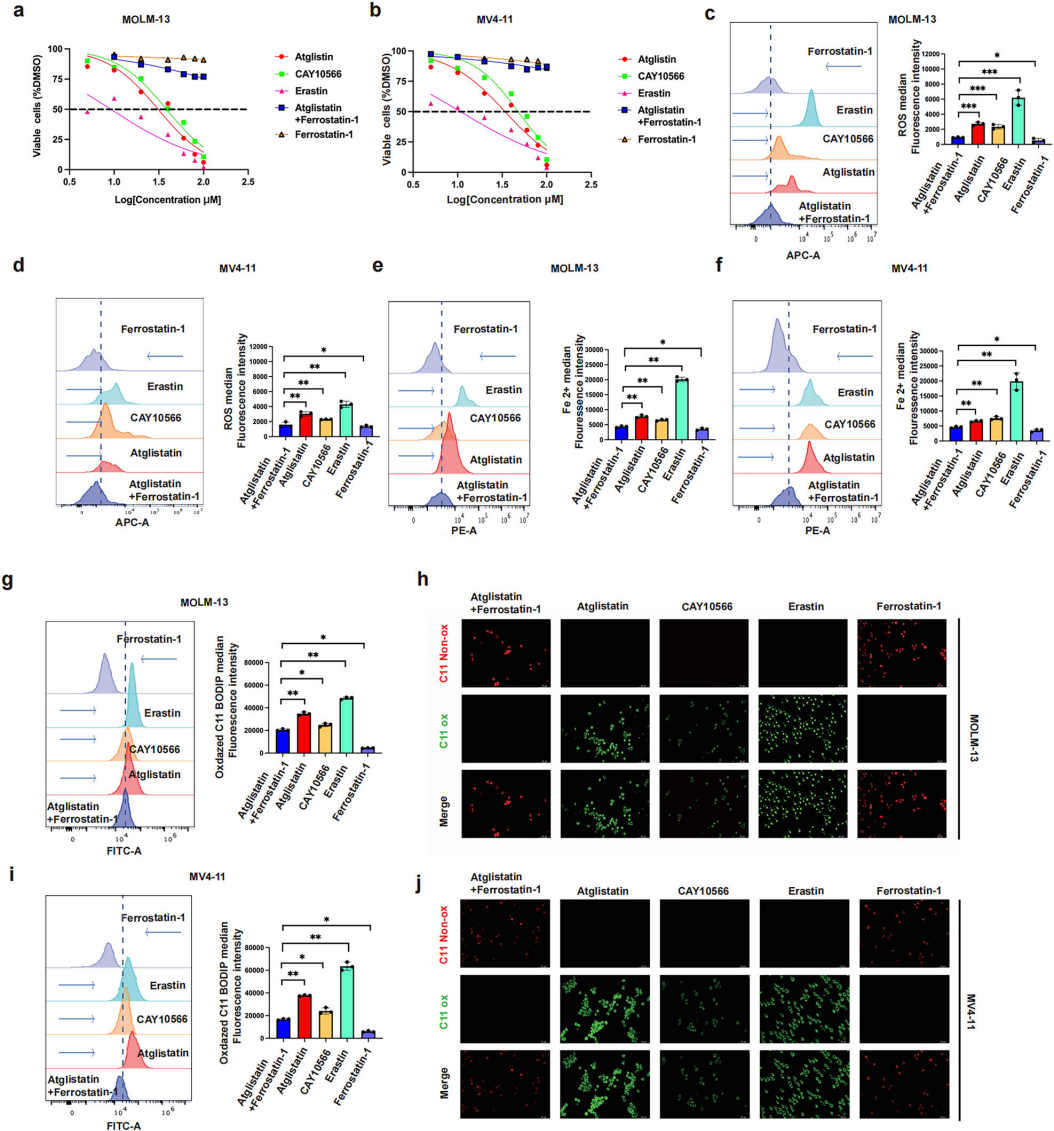
Comparison of ferroptosis phenotype changes in MOLM-13 and MV4-11 cells following treatment with ATGL inhibitor (atglistatin, 60 μM), SCD1 inhibitor (CAY10566, 10 nM), ferroptosis activator (Erastin, 5 μM), and ferroptosis inhibitor (Ferrostatin-1, 10 μM). **a** and **b** Cell viability was assessed 24 h after adding atglistatin, CAY10566, erastin, or ferrostatin-1 to MOLM-13 and MV4-11 cells. **c–j** ROS index, Fe²⁺ content, and C11-BODIPY581/591 changes in MOLM-13 and MV4-11 cells treated with Atglistatin, CAY10566, Erastin, or Ferrostatin-1 for 24 h. *P < 0.05; **P < 0.01; ***P < 0.001; ns not significant.

We reviewed the literature to determine how ATGL promotes SCD1 transcription and found that CEBPα can regulate *SCD1* in AML cells [11]. We constructed a diagram of the proposed mechanism of ATGL in AML (Fig. 4h). RT‒qPCR and Western blot analyses of MOLM-13 and MV4-11 cells were performed. Knockdown of *ATGL* reduced *CEBPα* expression and protein levels, whereas the overexpression of *ATGL* increased the expression and protein levels of *CEBPα* (Fig. 4i–k). Furthermore, the overexpression of *CEBPα* increased the expression and protein levels of SCD1 in MOLM-13 and MV4-11 cells in which *ATGL* was knocked down, but *ATGL* expression and protein levels were still decreased (Fig. 4l–o). Thus, ATGL could activate SCD1 transcription via CEBPα and inhibit ferroptosis. To further demonstrate that ATGL knockdown activates ferroptosis in cells, we administered five drug groups in MOLM-13 and MV4-11 cells: Commercial ATGL inhibitor (atglistatin, 60 μM) combined with ferroptosis inhibitor (ferrostatin-1, 10 μM), Commercial ATGL inhibitor (atglistatin, 60 μM), Ferroptosis activator (erastin, 5 μM, Commercial SCD1 inhibitor (CAY10566, 10 nM), Ferroptosis inhibitor (ferrostatin-1, 10 μM). After 24 h, cell viability assays were conducted, and the results indicated that atglistatin, erastin, and CAY10566 reduced cellular viability compared to the ferrostatin-1 or atglistation combined with ferrostation-1 (Fig. 5a and b). In parallel, the ROS index, Fe^2+^ levels, and lipid peroxidation were measured. The results indicated that compared with ferrostatin-1 or atglistation combined with ferrostatin-1, atglistatin, erastin, and CAY10566 increased the ROS index, Fe²⁺ content, and lipid peroxidation (Fig. 5c–j). Direct observation under a fluorescence microscope (×20) revealed markedly increased lipid peroxidation in the atglistatin, erastin, and CAY10566 groups compared with the ferrostatin-1 or atglistation combined with ferrostatin-1 groups (Fig. 5h, j). Furthermore, we also compared other cell death pathways in MOLM-13 and MV4-11 cells, and the results indicated that ferroptosis was still the main pathway responsible for the death of these cells (Fig. S5). These findings indicate that ATGL regulates ferroptosis in AML cells via the CEBPα/SCD1 axis.

### The synergistic effects of ATGL and FLT3 inhibitors suggest a new therapy for patients with *FLT3*-ITD-mutated AML and refractory and relapsed *FLT3*-ITD-mutated AML

**Fig. 6:**
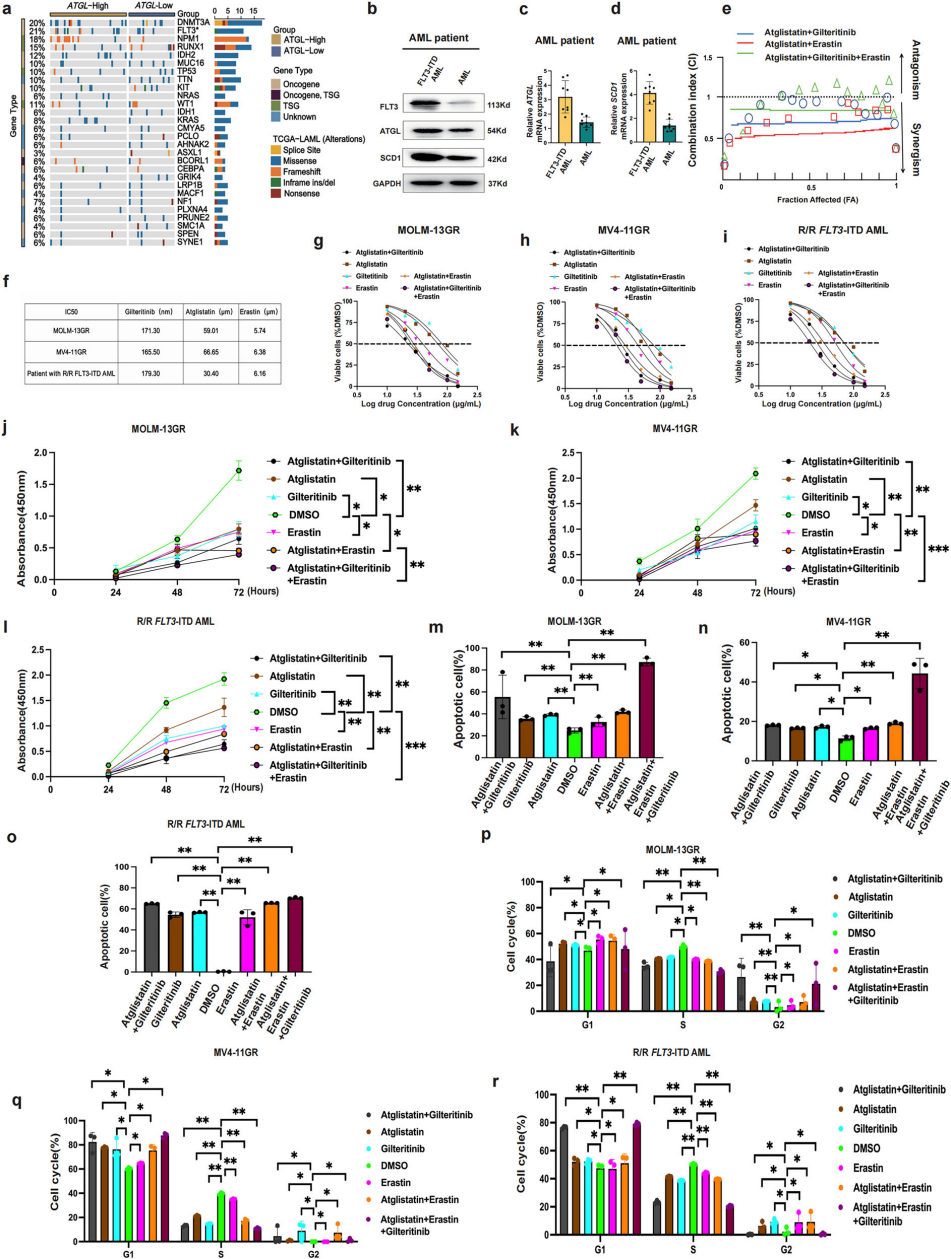
The synergistic effect of ATGL-inhibitor and FLT3-inhibitor in *FLT3*-ITD-mutated AML. **a** ATGL ratio of AML mutant genes in the TCGA database. **b–d** expression of ATGL, SCD1 in patient with *FLT3*-ITD-mutated AML cells and patient with AML cells was assessed by using Western blotting and RT- qPCR, GAPDH as an internal reference. **e** Chou-Syn software program was used to calculate FA (affected score) and CI (composite index) values (Atglistatin and Giltertinib, Atglistatin and Erastin, Atglistatin, Giltertinib and Erastin). **f** IC_50_ of FLT3-inhibitor (Gilteritinib), Erastin and Atglistatin in MOLM-13GR, MV4-11GR and a patient with R/R *FLT3*-ITD-mutated AML. **g–i** Comparison of dose–response curves derived from cell viability assays for monotherapy and combination therapy in MV4-11RG, MOLM-13GR, and a patient with R/R *FLT3*-ITD-mutated AML cells. The dashed line indicates the IC50 value. **j–l** Cell proliferation of MOLM-13GR, MV4-11GR, and a patient with R/R *FLT3-*ITD-mutated AML was detected by CCK8 at different time points (0, 24, 48, and 72 h) after treatment. **m–r** Confirmation of apoptosis and cell cycle after treatment using flow cytometry (representative images are shown). *P < 0.05; **P < 0.01; ***P < 0.001; ns not significant (Atglistatin 60 μM, gilteritinib 170 nM, erastin 5 μM)*P < 0.05; **P < 0.01; ***P < 0.001; ns not significant.

**Fig. 7:**
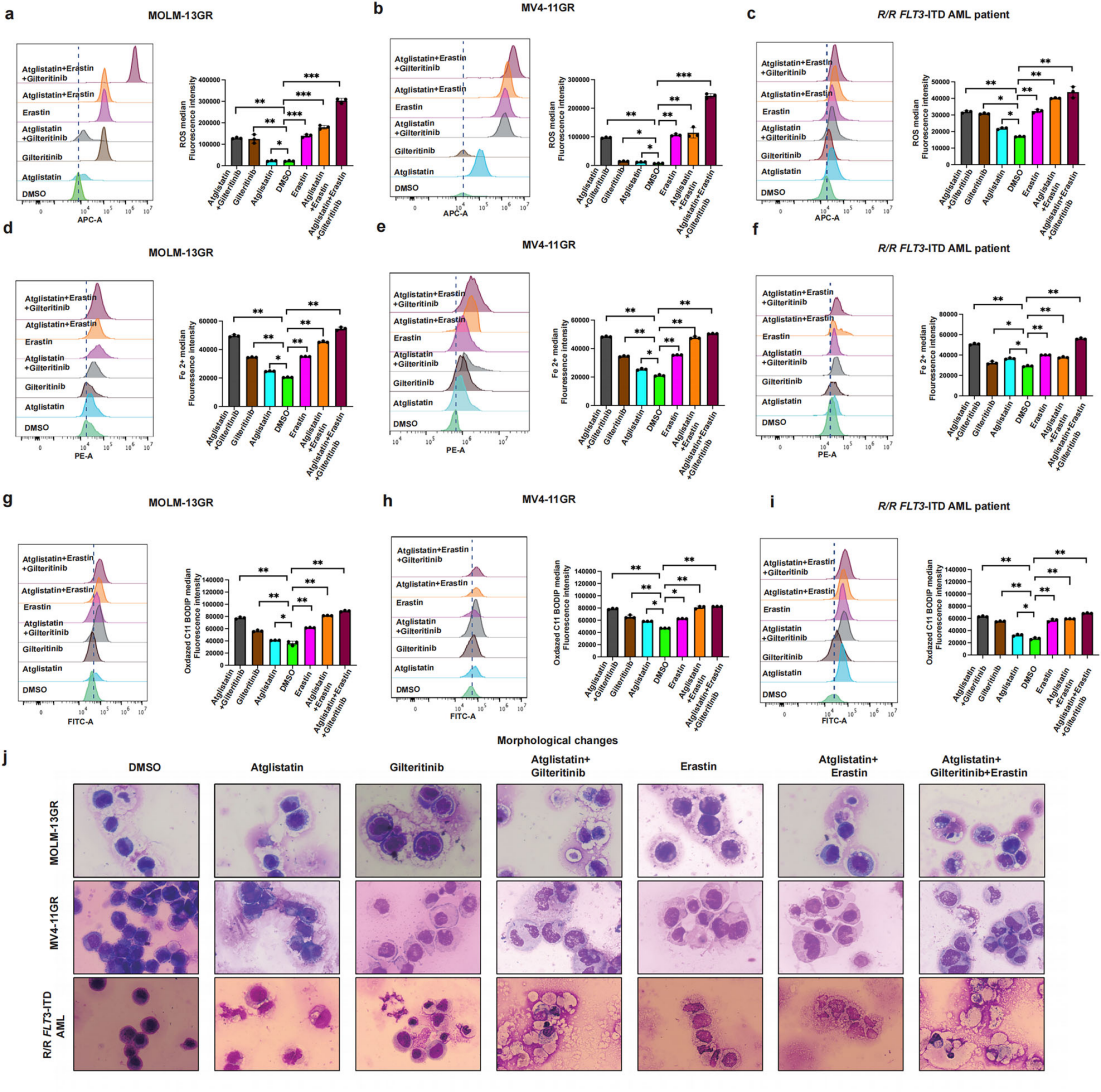
The changes of ferroptosis phenotype and cell morphology in different treatment groups were compared. **a**–**i** ROS index, Fe^2+^ content and C11-BODIPY581/591 changes at 24 h, Atglistatin (60 μM), gilteritinib (170 nM), and erastin(5 μM) in MOLM-13GR, MV4-11GR and R/R FLT3-ITD-mutated AML cells alone and in combination. **j** Cell morphology changes (40×) after treatment of the synergistic effect of ATGL-inhibitor and FLT3-inhibitor in patients with R/R FLT3-ITD-mutated AML cells, Atglistatin (60 μM), gilteritinib (170 nM), and erastin (5 μM). *P < 0.05; **P < 0.01; ***P < 0.001; *ns* not significant.

*FLT3* gene mutations account for 30% of AML cases and are major factors associated with poor treatment outcomes and a poor prognosis. Through TCGA, we observed significantly higher *ATGL* expression in patients with AML carrying *FLT3*-ITD mutations (Fig. 6a). However, the occurrence of resistance to FLT3 inhibitors (gilteritinib) during treatment can influence the final therapeutic outcome. The inhibition of ATGL can overcome resistance to gilteritinib in AML cells or increase their efficacy and provide a new option for clinical treatment. We detected higher ATGL protein levels and expression in patients with *FLT3*-ITD-mutated AML compared to patients with AML (Figs. 6b–d and S6). Concurrently, we collected samples from patients with relapsed/refractory *FLT3*-ITD-mutated AML to validate gilteritinib resistance (Fig. S8). ATGL and SCD1 protein levels and expression were higher in patients with relapsed/refractory *FLT3*-ITD-mutated AML than in those who responded to gilteritinib treatment. We established the gilteritinib-resistant MOLM-13 (MOLM-13GR) and MV4-11 (MV4-11GR) cells and confirmed their resistance (Fig. S7). Compared with parental cells, resistant cells exhibited increased aggregation and a higher gilteritinib IC50 (Fig. S7a–c). The cell cycle analysis revealed significantly increased S-phase progression and increased CyclinE/CDK2 mRNA expression in resistant cells compared with parental cells (Fig. S7d–f). Western blotting demonstrated that ATGL and SCD1 protein levels were significantly higher in resistant cells than in parental cells (Fig. S7g and h). We subsequently used Compusyn software to calculate the synergistic effect of combination drug therapy, and the results suggested a synergistic effect of gilteritinib in combination with atglistatin on *FLT3*-ITD-mutated AML cells (Fig. 6e). IC50 values were calculated for gilteritinib, atglistatin, and erastin when they were administered alone to MOLM-13GR, MV4-11GR and R/R *FLT3*-ITD mutated AML cells (Fig. 6f). Moreover, dose–effect curves were constructed to assess the therapeutic effects of single and combination drugs on MOLM-13GR, MV4-11GR, and R/R *FLT3*-ITD mutated AML cells, and the results indicated that compared with monotherapy, atglistatin combined with either gilteritinib or erastin, or the combination of the three drugs increased the death of MOLM-13GR, MV4-11GR cells and *FLT3*-ITD mutated AML cells (Fig. 6g–i). Next, we performed a CCK8 assay to investigate cell proliferation in the seven groups. The results indicated that cell proliferation was significantly lower in the combination therapy group than in the monotherapy group (Fig. 6j–l). Flow cytometry was performed to assess apoptosis and the cell cycle, and the results revealed that the apoptosis of MOLM-13GR, MV4-11GR cells, and *FLT3*-ITD mutated AML cells in the combination treatment group was significantly higher than that in the single-drug treatment group (Figs. 6m–r and S9). Moreover, the S phase of the combination group was shorter (Fig. S10). Flow cytometry also indicated that the ROS index, lipid peroxide levels, and Fe^2+^ content were increased in the combination group, with the greatest increase observed in the group treated with atglistatin in combination with gilteritinib with or without erastin (Fig. 7a–i). Finally, we performed Wright’s staining to investigate cellular morphological changes under a microscope (×40) (Fig. 7j). The results demonstrated that the most pronounced disruption in cellular morphology occurred in the groups treated with atglistatin in combination with either gilteritinib or erastin. Atglistatin treatment exacerbated the disruption of the cell membrane in both the MOLM-13GR and MV4-11GR cells. In conclusion, significantly higher expression of *ATGL* was observed in patients with *FLT3*-ITD-mutated AML, especially in those with R/R *FLT3*-ITD-mutated AML (Fig. S8c). Treatment with an ATGL inhibitor with or without a ferroptosis activator (erastin) enhanced the antitumor activity of the FLT3 inhibitor (gilteritinib) in *FLT3*-ITD-mutated AML or R/R *FLT3*-ITD-mutated AML cells, thereby promoting ferroptosis signaling in AML cells.

### ATGL inhibitors in combination with gilteritinib or erastin synergistically inhibit *FLT3*-ITD-mutated AML in vivo

**Fig. 8:**
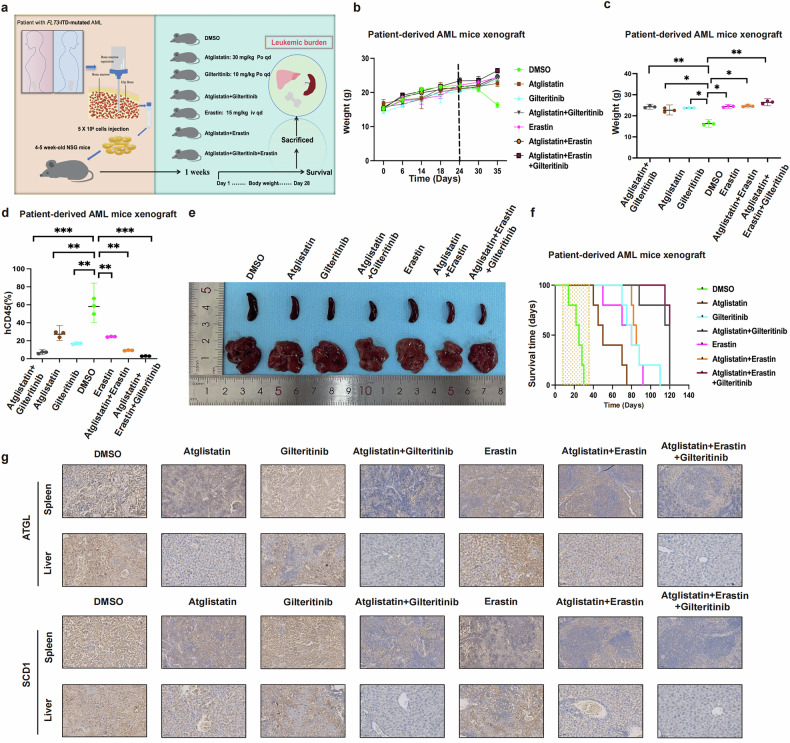
ATGL inhibitors in combination with gilteritinib or Erastin synergistically inhibit *FLT3*-ITD-mutated AML in vivo. **a** *FLT3*-ITD-mutated AML patient-derived tumor xenograft model and treatment flowchart, schematic diagram showing the protocol of the patient with *FLT3*-ITD-mutated AML-derived Xenograft Model. The treatment is for 28 days. The treatments in each group were Ctrl (DMSO), A-inhibitor (Atglistatin, 30 mg/kg, po, qd), Gilteritinib (10 mg/kg, po, qd), Combination-1 (Atglistatin + Gilteritinib), Erastin (15 mg/kg, iv, qd), Combination-2 (Atglistatin, Erastin), Combination-3 (Atglistatin, Gilteritinib, Erastin). **b** and **c** Body weight dynamics and final body weight in AML xenograft model over 35 Days. **d** and **e** AML xenograft model at 35 days after euthanasia, hCD45% leukemia burden, and spleen, liver morphology. **f** Survival curves of AML xenograft models after treatment for 120 days, which is the endpoint of observation. **g** Representative images of immunohistochemical staining for ATGL and SCD1 in spleen and liver excised from AML xenograft model (×40). *P < 0.05; **P < 0.01; ***P < 0.001; ns not significant.

Bone marrow samples were collected from patients with *FLT3*-ITD-mutated AML, and *FLT3*-ITD-mutated AML patient-derived tumor xenograft models were constructed by injecting 5 × 10^6^ AML cells into the tail vein of 4–5-week-old NSG mice (7 groups, *n* = 3) to further investigate the efficacy of the combination therapy in vivo (Fig. 8a). Different treatment protocols were used for the mice in the different groups 1 week after the AML cells were injected. The body weights of the mice were recorded every 2 days, and the changes in body weight were plotted (Fig. 8b). Comparisons of the body weights of the mice in each group at the end of the treatment revealed that the body weights of the mice in the combined treatment group were significantly greater than those of the mice in the control group (Fig. 8c). The mice were sacrificed at the end of 28 days of treatment, and bone marrow cells were extracted from the femur. Flow cytometry was performed to assess the hCD45% and revealed that the hCD45% of the combination therapy group was significantly lower than that of the control and single-agent groups (Fig. 8d). Notably, the hCD45% value was lowest in the group treated with atglistatin combined with gilteritinib and erastin. Measurements of the sizes of the spleens and livers of the mice revealed that the spleens of the mice in the combination treatment group were smaller than those of the mice in the single-drug and control groups, especially in the triple-drug combination therapy group (Fig. 8e). However, no significant differences were observed in the size of the liver. Another set of experiments (7 groups; *n* = 5) was conducted under the same conditions to compare the survival times of the mice in different treatment groups, with 120 days as the observation endpoint. Notably, the outcomes of the combination treatment group were superior to those of the control and monotherapy groups (Fig. 8f). We established an allogeneic transplant mouse model using fluorescent MV4-11GR (F-Luc) cells, employing the same experimental methods and groupings as described above, and directly observed changes in the leukemic burden during treatment using fluorescence in vivo imaging to more intuitively assess treatment efficacy in AML mice (Fig. S11). The results indicated that all the combination regimens induced enhanced tumor cell-killing effects, with fluorescent in vivo imaging directly demonstrating the complete disappearance of leukemia burden in AML allogeneic transplant mice when the three drugs were used in combination (Fig. S11g). Finally, we performed immunohistochemistry to compare the level of ATGL and SCD1 in the liver and spleen (Figs. 8g and S11h). ATGL and SCD1 were significantly decreased in the liver and spleen of mice in the combination treatment groups compared with the single-agent treatment groups. These results collectively demonstrate that atglistation combined with gilteritinib or erastin constitutes an effective therapeutic regimen in the AML xenograft model compared to monotherapy.

## Discussion

Lipid metabolism, a component of metabolic reprogramming, provides energy for tumor progression and maintains the energy supply required for tumor cell proliferation [23, 24]. Research by Ferrer et al. on leukemia stem cells and their microenvironment revealed that the microenvironment enhances tumor cell energy production through the activation of the tricarboxylic acid (TCA) cycle and oxidative phosphorylation (OXPHOS) and by influencing reactive oxygen species (ROS) levels. These processes promote leukemia progression, chemotherapy resistance, and immunotherapy resistance [25, 26]. This study demonstrated that ATGL not only serves as a diagnostic and prognostic marker for patients with AML but also has therapeutic potential. The high energy demands of cancer cells typically suppress ROS production, activating antioxidant defense mechanisms to prevent cellular damage [27, 28]. Previous studies revealed inconsistent patterns of *ATGL* expression across different tumors, with varying disease associations. Elevated ATGL levels in colon cancer stem cells promote lipid metabolism and tumorigenic reprogramming. In breast cancer, eliminating ATGL activity increases natural killer cell activity in vivo and reduces lung metastasis [29, 30]. However, targeting ATGL as a therapeutic modulator promotes metabolic plasticity in patients with advanced prostate cancer [31]. Our study identifies ATGL as a key lipid regulator in AML and elucidates its mechanism of action, thereby identifying therapeutic strategies for AML.

Transcriptome sequencing revealed that ATGL inhibition in AML tumor cells differentially affects fatty acid catabolism, the tricarboxylic acid cycle, glucose metabolism, cysteine and glutamate metabolism, apoptosis, and ferroptosis. AML cells can utilize fatty acids as a biofuel, but fatty acid oxidation induces ferroptosis [32, 33]. However, due to funding and time constraints, we could not assess the contributions of different signaling pathways in this study. Concurrently, ATGL influenced G1, S, and G2 phases of AML cells. These observations may stem from the role of ATGL in promoting cell proliferation during the proliferative phase by stimulating membrane lipid biosynthesis. These observations correlate with the significant disruption of AML cell membranes observed when ATGL inhibitors were used as therapeutic agents. In this study, ATGL inhibition reduced *SCD1* expression, promoted reactive oxygen species (ROS) production, increased Fe²⁺ levels, and increased lipid peroxide generation. Mechanistically, these findings confirm that ATGL regulates SCD1 transcription and its biological effects. As a key protein involved in lipid metabolism, SCD1 has been shown to regulate malignant ascites formation and metastasis in ovarian cancer patients via the ferroptosis pathway [15]. Concurrently, SCD1 drives the development of drug resistance in gastric cancer patients through ferroptosis [34]. These findings, combined with our findings, suggest that the relationship between SCD1 and drug resistance may arise through the regulatory effects of ATGL [35]. Notably, the efficacy of commercially available ATGL and SCD1 inhibitors is equivalent. Furthermore, a literature review revealed that SCD1 is regulated by CEBPα in *FLT3*-ITD-mutated AML. Our study further confirms that ATGL positively regulates CEBPα, revealing for the first time how ATGL inhibits ferroptosis at the transcriptional level via the CEBPα/SCD1 axis [36, 37], thereby accelerating AML progression and elucidating the mechanism of action of ATGL in AML. However, the precise relationship between ATGL and CEBPα will be addressed in future studies.

In AML treatment, *FLT3* mutations lead to a poor prognosis and poor therapeutic response. Resistance to the commonly used FLT3 inhibitor gilteritinib also develops during long-term monotherapy. This research revealed a significant increase in *ATGL* expression in patients with *FLT3*-ITD-mutated AML. By constructing gilteritinib-resistant cell lines, we confirmed that ATGL inhibitors, with or without concurrent treatment with ferroptosis activators, exert synergistic anti-*FLT3*-ITD-mutated AML effects when they are coadministered with gilteritinib. By modulating fatty acid oxidation, ATGL inhibitors increase the death of AML cells [38–40]. Our research confirms that ATGL inhibitors represent a novel therapeutic option for patients with *FLT3*-ITD-mutated AML or relapsed/refractory *FLT3*-ITD-mutated AML by increasing the sensitivity of AML cells to gilteritinib. Additionally, ATGL inhibitors may emerge as therapeutic agents for AML.

Together, these findings collectively reveal that ATGL regulates ferroptosis in AML through redox responses mediated by the CEBPα/SCD1 axis, highlighting the role of ATGL in AML progression at the transcriptional level. Based on our results, ATGL may emerge as a promising therapeutic target or checkpoint in AML. Furthermore, in gilteritinib-resistant *FLT3*-ITD-mutated AML, ATGL inhibitors can reverse drug resistance. Although ATGL inhibitors have not yet entered clinical trials, this combination therapy represents a safe and effective novel treatment strategy for patients with *FLT3*-ITD-mutated AML and relapsed/refractory *FLT3*-ITD-mutated AML.

## Supplementary information

Supplemental material

Original Data

## Data Availability

The raw data supporting the conclusions of this manuscript will be made available by the author, without undue reservation, to any qualified researcher.
