# Supplementary material for: ATGL suppresses ferroptosis in acute myeloid leukemia cells by modulating the CEBPα/SCD1 axis and induces gilteritinib resistance
Source: Cell Death Dis. 2026 Jan 9;17(1):171. doi: 10.1038/s41419-025-08388-0 (PMC12876964; doi:10.1038/s41419-025-08388-0)
Supplement: Supplementary file 1 — Supplemental material [file 41419_2025_8388_MOESM1_ESM.docx]

| **Supplementary materials** | | |
| --- | --- | --- |
| Gene |  | |
| Sh*ATGL*-1 | GUGCCAAGUUCAUUGAGGUTT | |
| Sh*ATGL*-2 | ACCUCAAUGAACUUGGCACTT | |
|  | | |
| Gene | Forward primer 5’➡3’ | Reverse primer 5’➡3’ |
| *PI4KB* | CCTGCTCAACCATAAGCTCCC | AGTTTTCTACGGACCTCGTACT |
| *SREBF2* | CCTGGGAGACATCGACGAGAT | TGAATGACCGTTGCACTGAAG |
| *ATGL* | GGCTTCCTCGGCGTCTACTA | TTTACCAGGTTGAAGGAGGG |
| *STARD7* | GTCTTCGTTTGGGACGAGGAG | CTTCCAACCGCTTCATCTCATT |
| *CPNE3* | GTCAGACCCTTTATGTGTGTTGT | TGGAAAATTGGGGATTCAAGCAA |
| *PNPLA4* | GTGGGATGGAGTCGATTCTTCC | CCACTAGCTTCAGTCCTGCATA |
| *MED20* | ATGGGAGTGACTTGTGTGTCC | GCATCTCCAATTTCCGGGTAAG |
| *MAPKAPK2* | CGCAGTTCCACGTCAAGTC | GGGCGAATTTCTCCTGGGTC |
| *GPD1L* | CCCCTGAAAGTGTGCATCGT | GGCAGCTTGTGTCCAGGAA |
| *PMVK* | CCTTTCGGAAGGACATGATCC | TCTCCGTGTGTCACTCACCA |
| *DEGS1* | GAGATCCTGGCAAAGTATCCAGA | CAAACGCATAGGCCCCAAA |
| *ACER3* | ACTACTCCGTGACCTGGTACA | GCACCGAACATTGGAGGTATAAT |
| *SLC25A1* | CCGTCAGGTTTGGAATGTTCG | TAACCCCGTGGAAGAATCCTC |
| *ECHS1* | TGAGCTTGCCATGATGTGTGA | AACAGGACAAATCTTGCTGACA |
| *TGFB1* | GGCCAGATCCTGTCCAAGC | GTGGGTTTCCACCATTAGCAC |
| *ARF3* | ATGGGCAATATCTTTGGAAACCT | TGAACCCAATGGTAGGGATGG |
| *INPP5F* | TAGCTTGACCTATGACCTGACC | CTGCTCTACGAGCTGTGAGT |
| *SLC44A1* | GGACCGTAGCTGCACAGAC | GCCACAAATAAATCCCATCCCA |
| *GPX1* | CAGTCGGTGTATGCCTTCTCG | GAGGGACGCCACATTCTCG |
| *PLA2G4A* | TACCAGCACATTATAGTGGAGCA | GCTGTCAGGGGTTGTAGAGAT |
| *SCD1* | ATGGATATCGCCCCTACGAC | GATGTGCCAGCGGTACTCAC |
| *CEBPα* | CTGGAGCTGACCAGTGACAA | GAGACCCTGAGACCCGAAAC |
| **Table. S1. Related sequence** | | |

| **ID** | **Gender** | **Age**  **(Years)** | **Disease status** | **FAB subtype** | **Mutuation Gene** | **FLT3 mutuation ratio** |
| --- | --- | --- | --- | --- | --- | --- |
| **AML-1** | Female | 51 | Relapse | M1 | *FLT3*-ITD, NARS | 56.25% |
| **AML-2** | Female | 51 | Relapse | M4 | *FLT3*-ITD | 78.83% |
| **AML-3** | Male | 72 | Relapse | M1 | *FLT3*-ITD | 70.23% |
| **AML-4** | Male | 51 | Relapse | M2 | *FLT3*-ITD, NPM1 | 55.77% |
| **AML-5** | Female | 73 | Relapse | M3 | *FLT3*-ITD, CEBPA | 62.84% |
| **AML-6** | Male | 24 | Relapse | M5 | *FLT3*-ITD | 44.35% |
| **AML-7** | Female | 67 | Relapse | M5 | *FLT3*-ITD | 46.39% |
| **AML-8** | Male | 25 | Relapse | AML | *FLT3*-ITD | 44.35% |
| **AML-9** | Female | 53 | Relapse | M6 | *FLT3*-ITD | 23.45% |
| **AML-10** | Male | 64 | Relapse | M5 | *FLT3*-ITD | 47.89% |
| **AML-11** | Female | 58 | Relapse | M4 | *FLT3-*ITD | 57.89% |
| **AML-12** | Male | 62 | Relapse | M5 | *FLT3*-ITD | 78.92% |
| **AML-13** | Male | 48 | First diagnosis | M4 | *FLT3*-ITD | 43.27% |
| **AML-14** | Female | 65 | First diagnosis | M4 | *FLT3*-ITD | 56.43% |
| **AML-15** | Male | 67 | First diagnosis | M5 | *FLT3*-ITD | 48.92% |
| **AML-16** | Female | 62 | First diagnosis | M4 | *FLT3-*ITD | 55.67% |
| **AML-17** | Male | 54 | First diagnosis | M5 | *FLT3*-ITD | 62.44% |
| **AML-18** | Female | 71 | First diagnosis | AML | *FLT3*-ITD | 66.51% |
| **AML-19** | Male | 53 | First diagnosis | M3 | *FLT3*-ITD | 23.45% |
| **AML-20** | Female | 55 | First diagnosis | M4 | *FLT3*-ITD | 71.20% |
| **AML-21** | Male | 60 | First diagnosis | M2 | *FLT3*-ITD | 45.66% |
| **AML-22** | Male | 74 | First diagnosis | M2 | NA | NA |
| **AML-23** | Male | 72 | First diagnosis | M2 | NA | NA |
| **AML-24** | Male | 54 | First diagnosis | M2 | NA | NA |
| **AML-25** | Male | 42 | First diagnosis | M4 | NA | NA |
| **AML-26** | Female | 67 | First diagnosis | M7 | NA | NA |
| **AML-27** | Male | 49 | First diagnosis | M1 | NA | NA |
| **AML-28** | Female | 64 | First diagnosis | M2 | NA | NA |
| **AML-29** | Female | 74 | First diagnosis | M1 | NA | NA |
| **Table. S2. Patient information** | | | | | | |

| \| **Inhibitor, drug** \| **References** \| \| --- \| --- \| \| **Atglistatin,**  **60µm** \| Zhang Q, Shen X, Yuan X, et al.Lipopolysaccharidebinding protein resists hepatic oxidative stressbyrequlating lipid droplet homeostasis.Nature Communications.2024.15(1): 3213 \| \| **CAY10566,**  **10 nM** \| Xuan Y, Wang H, Yung MM, Chen F, Chan Ws, Chan YS, Tsui SK, Ngan HY, Chan KK, Chan DWSCD1/FADS2 fatty acid desaturases equipoise lipid metabolic activity and redox-driven ferroptosis in ascitesderived ovarian cancer cells.Theranostics.2022 Apr 24:12(7 ):3534-3552. \| \| **ferrostatin-1,**  **10 µm** \| Xuan Y, Wang H, Yung MM, Chen F, Chan Ws, Chan Ys, Tsui SK, Ngan HY, Chan KK, Chan DW.SCD1/FADS2 fatty acid desaturases equipoise lipid metabolic activity and redox-driven ferroptosis in ascitesderived ovarian cancer cells. Theranostics. 2022 Apr 24;12(7 ):3534-3552. \| \| **Erastin,**  **5 uM** \| Yang Y, Luo M, Zhang K, Zhang J, Gao T, Connell DO, Yao F, Mu C, Cai B, Shang Y, Chen W. Nedd4ubiquitylates VDAC2/3 to suppress erastin-induced ferroptosis in melanoma. Nat Commun. 2020 Jan23:11(1):433. \| \| **Caspase Inhibitor,**  **60 nM** \| Lee D, et, al. Potent and selective nonpeptide inhibitors of caspases 3 and 7 inhibit apoptosis and maintaincell functionality.J Biol Chem. 2000 May 26:275(21):16007-14. \| \| **Necrostatin-5,**  **240 nM** \| Gonzalez-Juarbe N, Gilley RP,Hinoiosa CA, et al.Pore-Forming Toxins Induce MacrophageNecroptosisduring Acute Bacterial Pneumonia. PLoS Pathog.2015:11(12):e1005337. \| \| **BafA1,**  **5 nM** \| Du L. Wu Y, Han X, et al.NICE-3-knockdowrinduces cell cycle arrest and autophagy in lung adenocarcinomacells via the AKT/mTORC1 signalingpathway. Experimental and Therapeutic Medicine.2021 ,21(6):1-8 \| \| **Atglistatin,**  **30 mg/kg** \| Mayer N, et al. Development of small-molecule inhibitors targeting adipose triglyceride lipase. (2013) NatChem Biol. 9(12):785-7 \| \| **Gilteritinib,**  **10 mg/kg** \| Hu C, Zhang Y, Yang J, et al.Ningetinib, a novelFLT3 inhibitor, overcomes secondary drug resistancein acutemyeloid leukemia.Cell Communication andSignaling.2024.22(1):1-14. \| \| **Erastin,**  **15 mg/kg** \| Chen C.Yang Y, Guo Y. et al.CYP1B1 inhibitsferroptosis and induces ant-PD-1 resistance bydegradingACSL4 in colorectal cancer.Cell Death &Disease.2023.14(4): 271. \| |
| --- | --- | --- | --- | --- | --- | --- | --- | --- | --- | --- | --- | --- | --- | --- | --- | --- | --- | --- | --- | --- | --- | --- |
| **Table. S3. Concentrations of drugs or inhibitors used in the study and references** |

| \| **Antibody** \| **Concentration** \| \| --- \| --- \| \| **ATGL(WB)** \| 1:1000 \| \| **SCD1(WB)** \| 1:1000 \| \| **GAPDH(WB)** \| 1:1000 \| \| **Cleave-Caspase3(WB)** \| 1:1000 \| \| **Bax(WB)** \| 1:1000 \| \| **Bcl-2(WB)** \| 1:1000 \| \| **CEBPα(WB)** \| 1:500 \| \| **ATGL(ICH)** \| 1:100 \| \| **SCD1(ICH)** \| 1:100 \| |
| --- | --- | --- | --- | --- | --- | --- | --- | --- | --- | --- | --- | --- | --- | --- | --- | --- | --- | --- | --- | --- |
| **Table. S4. Concentration of antibodies used in the research** |

| 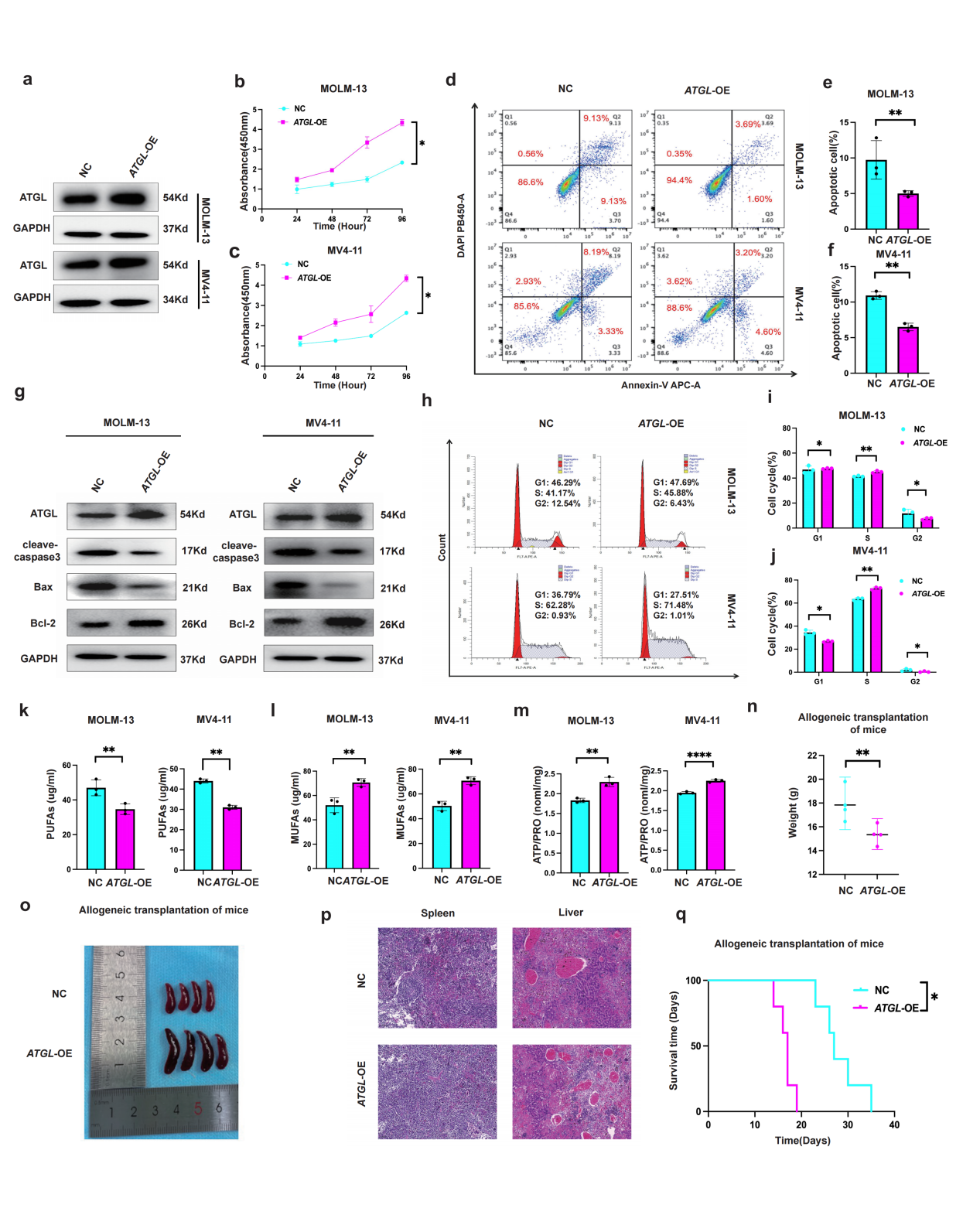 |
| --- |
| **Fig. S1 *ATGL* overexpression promotes malignant progression in AML cells in vivo and in vitro. a.** The efficiency of *ATGL* overexpression (*ATGL*-OE) by lentiviral vectors in MOLM-13 and MV4-11 cells was confirmed by Western bloting. **b-c.** Cell proliferation of MOLM-13 and MV4-11 cells was measured by CCK8 assay at different time points (0, 24, 48, 72 and 96 h) after *ATGL*-OE transduction. **d-g.** Apoptotic proteins (Bax, Bcl-2, Cleave-Caspase3) were measured by Western blot, and flow cytometry (representative images shown) was used to confirm apoptosis in *ATGL*-OE cells. h-j. Cell cycle changes in *ATGL*-OE cells tested using flow cytometry. **k-m.** Determination of polyunsaturated fatty acids, monounsaturated fatty acids, and ATP changes in MOLM-13, MV4-11 cells following *ATGL*-OE. **n.** Final body weight of AML mice. **o.** Spleen changes in *ATGL*-OE groups and control groups in AML mice. **p.** Pathological staining of spleen and liver from AML mice (40X). **q.** Comparison of survival curves in AML mice models. **P* < 0.05; ***P* < 0.01; ****P*< 0.001. |

| a | b |
| --- | --- |
| 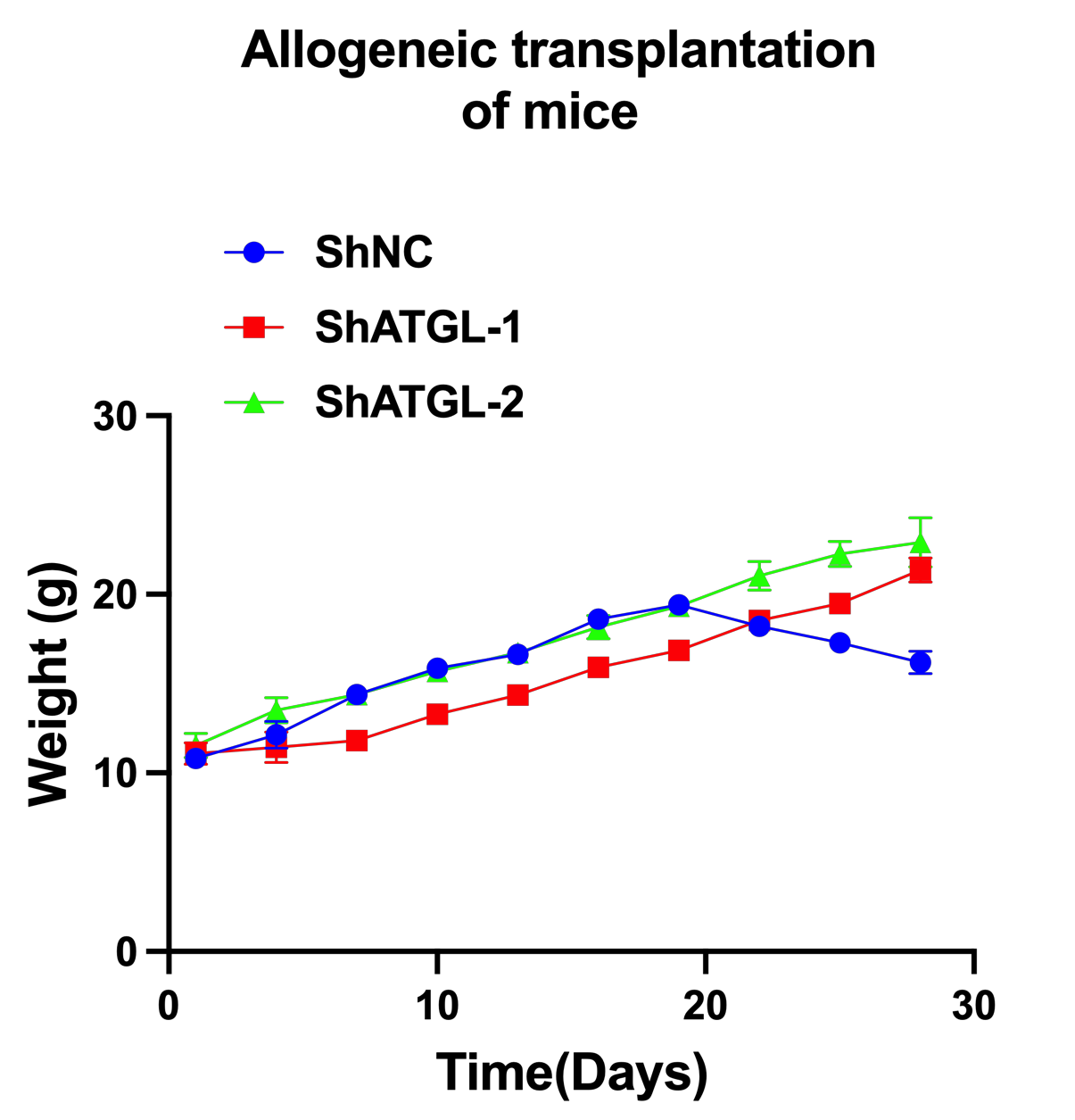 | 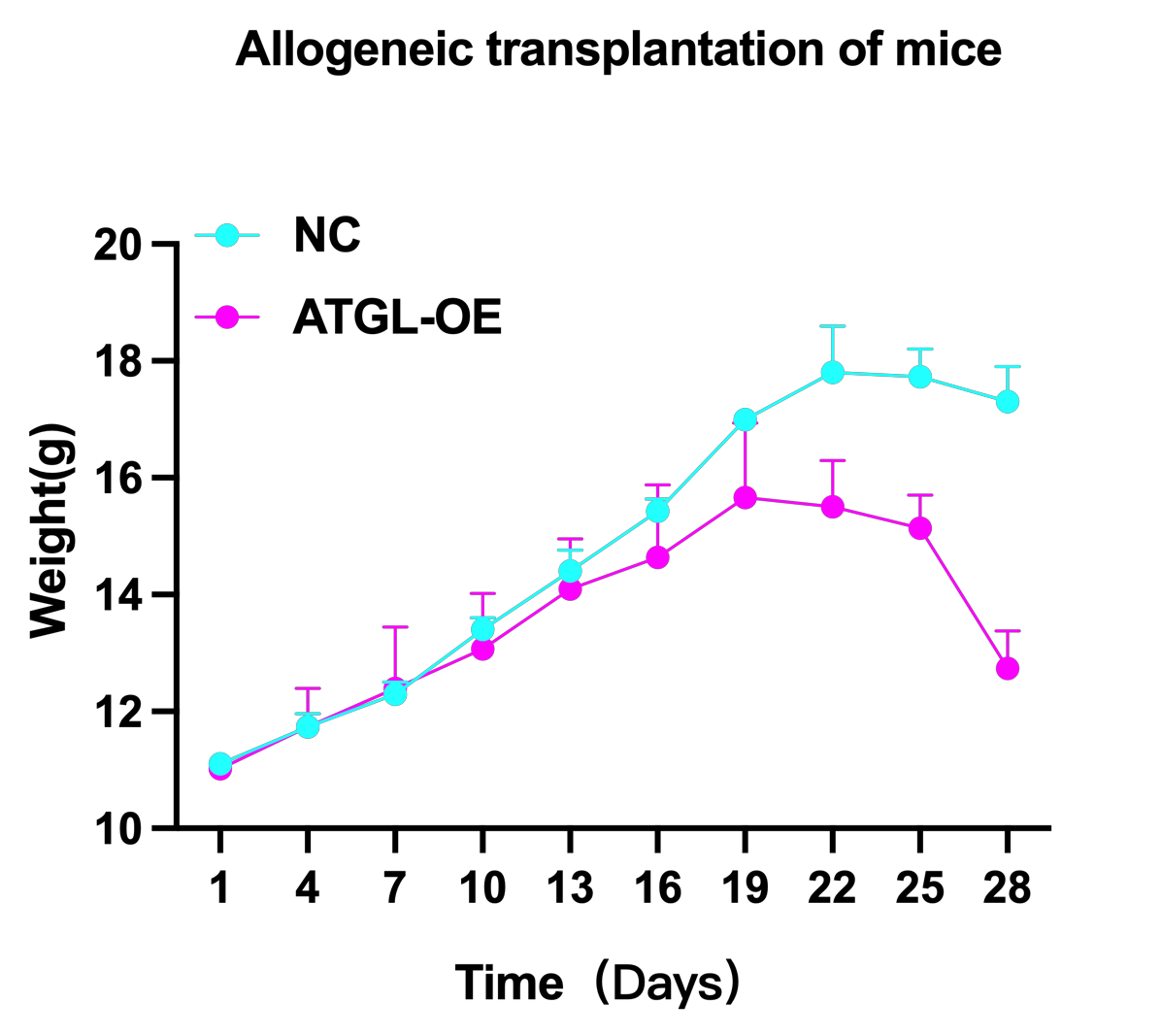 |
| **Fig. S2 AML xenograft model weight change.** a. Body weight changes of *ATGL* knockdown in AML xenograft model. b. Body weight changes of *ATGL* overexpression in AML xenograft model. | |

| 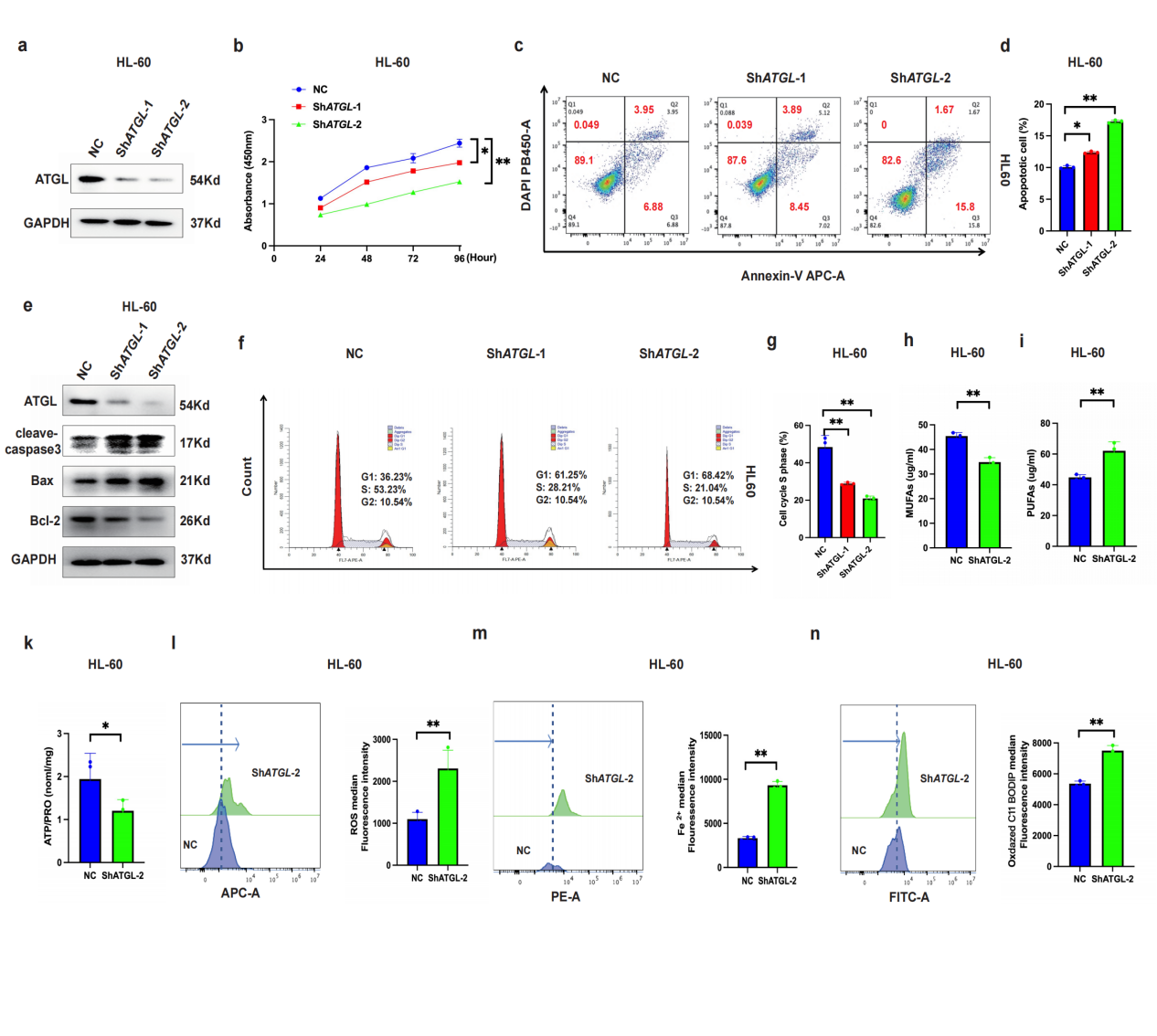 |
| --- |
| **Fig S3. Biological characteristics of *ATGL* knockdown in HL60 cells.** **a.** Western blotting detection of Sh*ATGL* knockdown efficacy in HL60 cells. **b.** CCK8 assay for cell growth assessment. **c-e.** Flow cytometry analysis of apoptotic changes and Western blotting of apoptotic protein alterations in *ATGL*-knockdown HL60 cells. **f-g.** Flow cytometry detection of cell cycle changes in HL60 cells. **h–k.** Changes in monounsaturated fatty acids, polyunsaturated fatty acids, and ATP levels in *ATGL*-knockeddown HL60 cells. **l–m.** Changes in ROS index, Fe²⁺ content, and lipid peroxide content in HL60 cells after *ATGL* knockdown. **P* < 0.05; ***P* < 0.01; ****P* < 0.001; ns, not significant. |

| 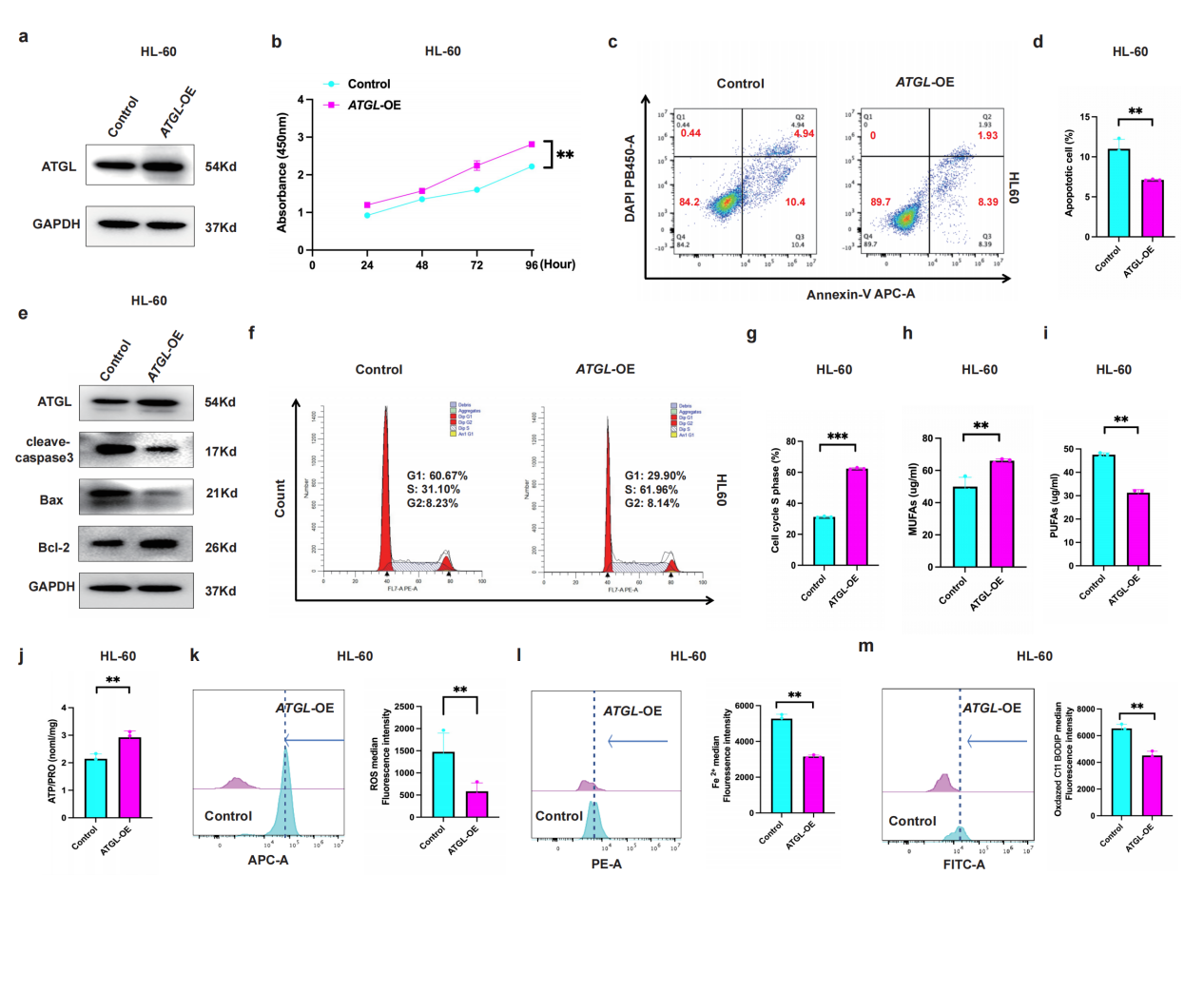 |
| --- |
| **Fig S4. Biological characteristics of *ATGL* overexpression in HL60 cells.** **a.** Western blotting detection of *ATGL*-overexpression efficacy in HL60 cells. **b.** CCK8 assay for cell growth assessment. **c-e.** Flow cytometry analysis of apoptotic changes and Western blotting of apoptotic protein alterations in *ATGL*-overexpression HL60 cells. **f-g.** Flow cytometry detection of cell cycle changes in HL60 cells. **h–j.** Changes in monounsaturated fatty acids, polyunsaturated fatty acids, and ATP levels in HL60 cells with *ATGL*-overexpression in HL60. k**–m.** Changes in ROS index, Fe²⁺ content, and lipid peroxide content in HL60 cells after *ATGL* overexpression. **P* < 0.05; ***P* < 0.01; ****P* < 0.001; ns, not significant. |

| 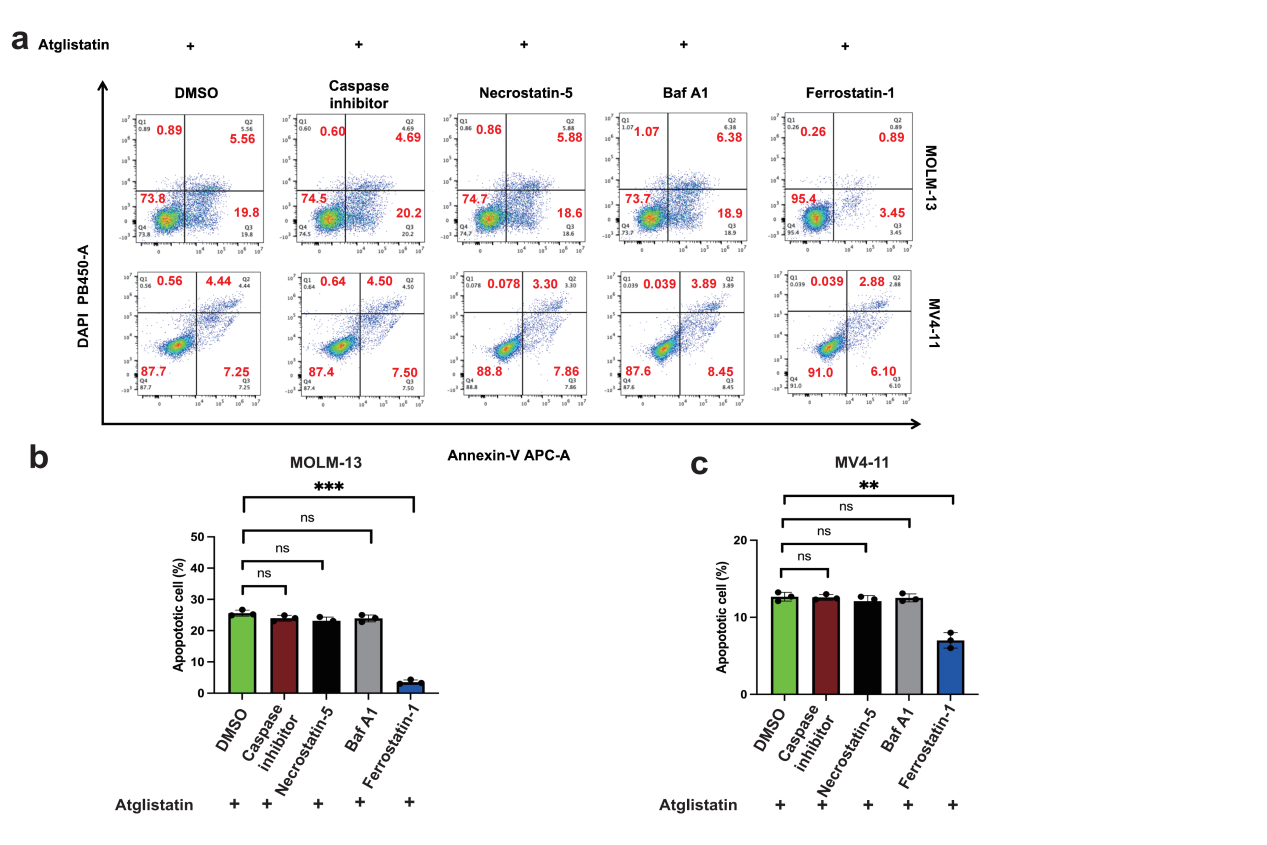 |
| --- |
| **Fig. S5 a. Effects of Atglstatin (60 μM) and other cell death Inhibitors (Caspase Inhibitor, 60 nM), necrostatin-5 (240 nM), bafA1 (5 nM), ferrostatin-1 (10 μM) on cell death in MOLM-13 and MV4-11 cells.** **P* < 0.05; ***P* < 0.01; ****P* < 0.001; ns, not significant. |

| 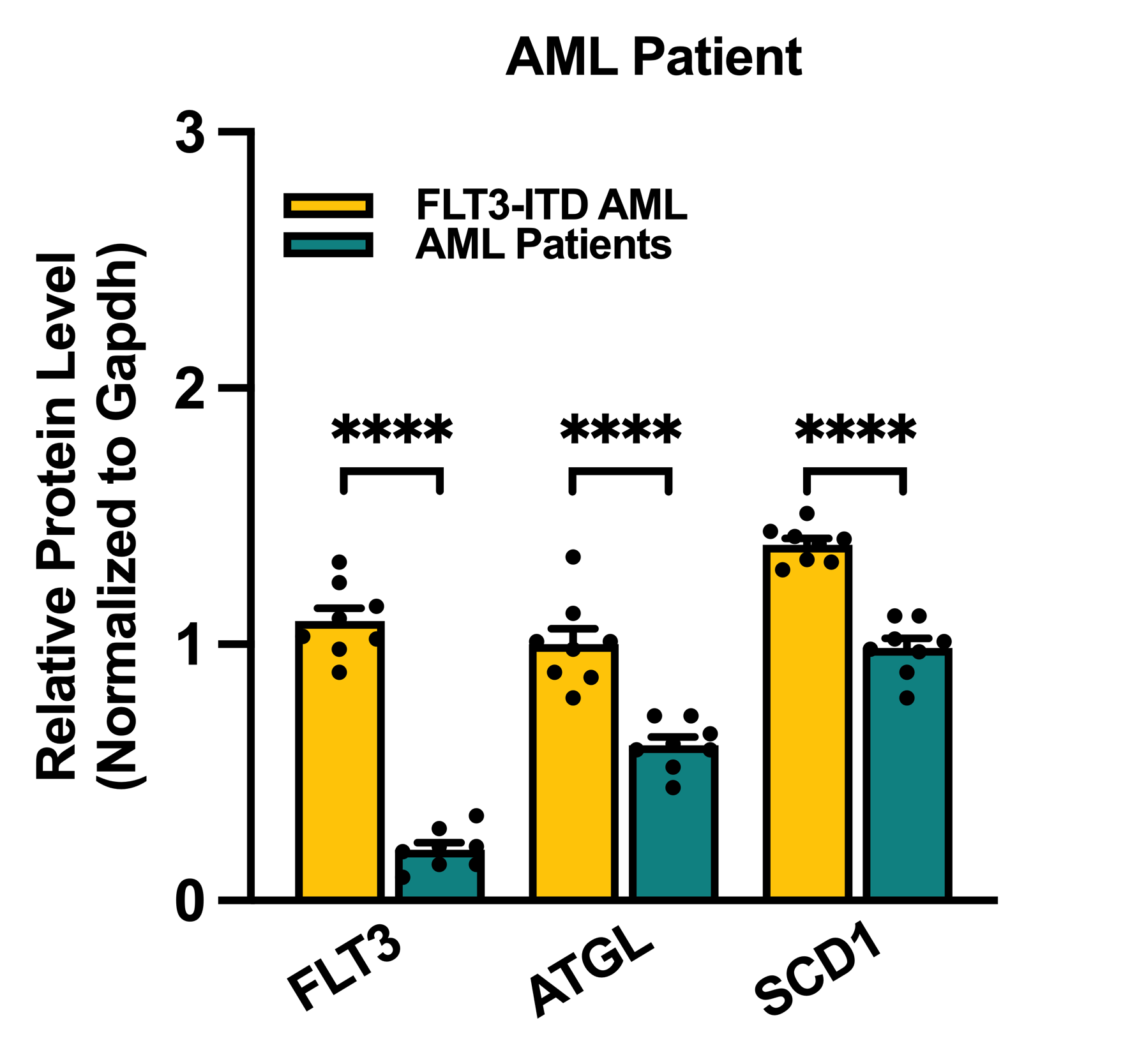 |
| --- |
| **Fig. S6 Western Blot Immunodetection Statistics for Patients of AML and *FLT3*-ITD-mutated AML.** **P* < 0.05; ***P* < 0.01; ****P* < 0.001; ns, not significant. |

| 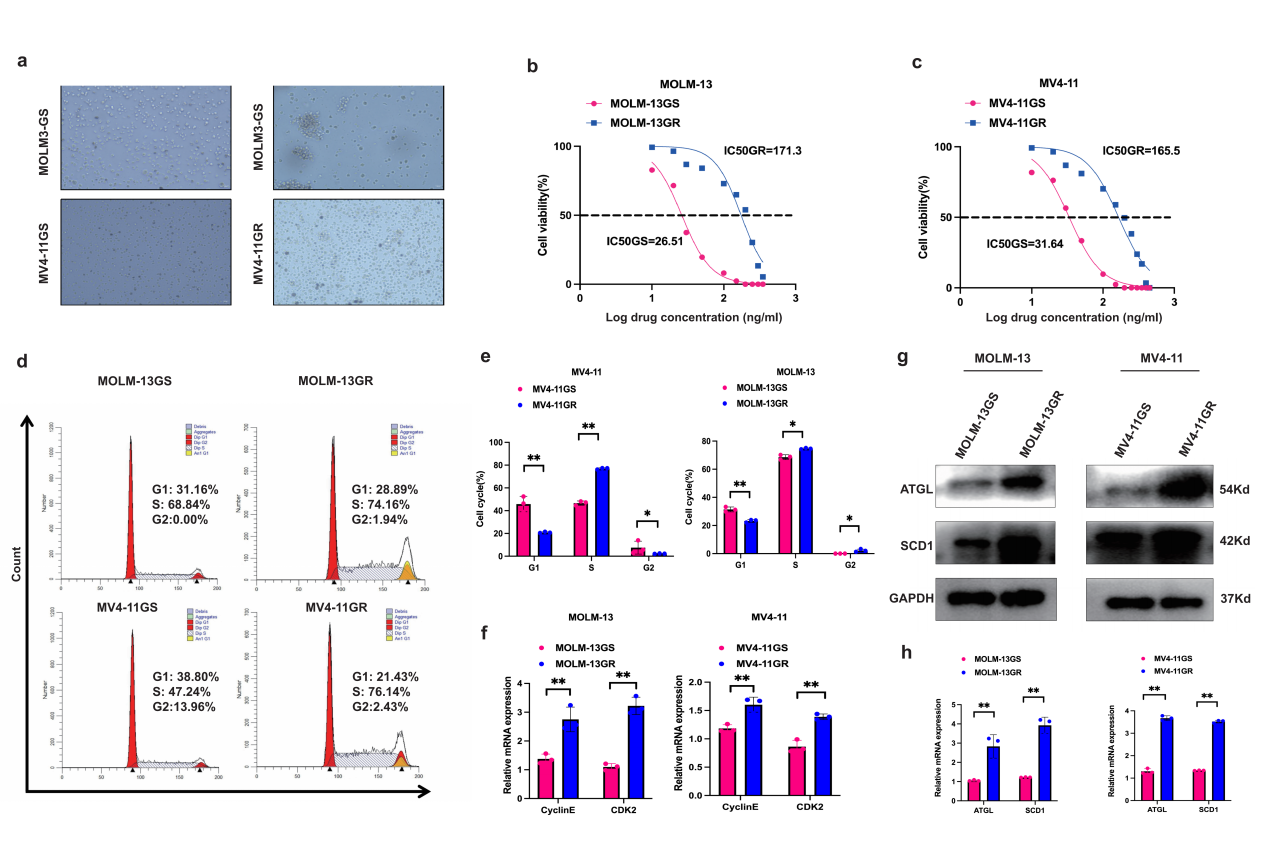 |
| --- |
| **Fig. S7 Construction of Gilteritinib-Resistant Cell Lines. a.** Microscopic morphology of MOLM-13 and MV4-11 gilteritinib-resistant cell lines and their parental cell lines. **b-c.** IC50 values of gilteritinib in gilteritinib-resistant MOLM-13 and MV4-11 cell lines versus parental MOLM-13 and MV4-11 cell lines. **d-e.** Cell cycle analysis of gilteritinib-resistant MOLM-13 and MV4-11 cells versus parental MOLM-13 and MV4-11 cell lines. **f.** RNA expression of cell cycle genes CyclinE and CDK2 in MOLM-13 and MV4-11 gilteritinib-resistant lines and MOLM-13 and MV4-11 parental lines. **g-h.** Expression of *ATGL* and *SCD1* in MOLM-13 and MV4-11 gilteritinib-resistant cell lines and MOLM-13 and MV4-11 parental cell lines. **P* < 0.05; ***P* < 0.01; ****P* < 0.001; ns, not significant. |

| 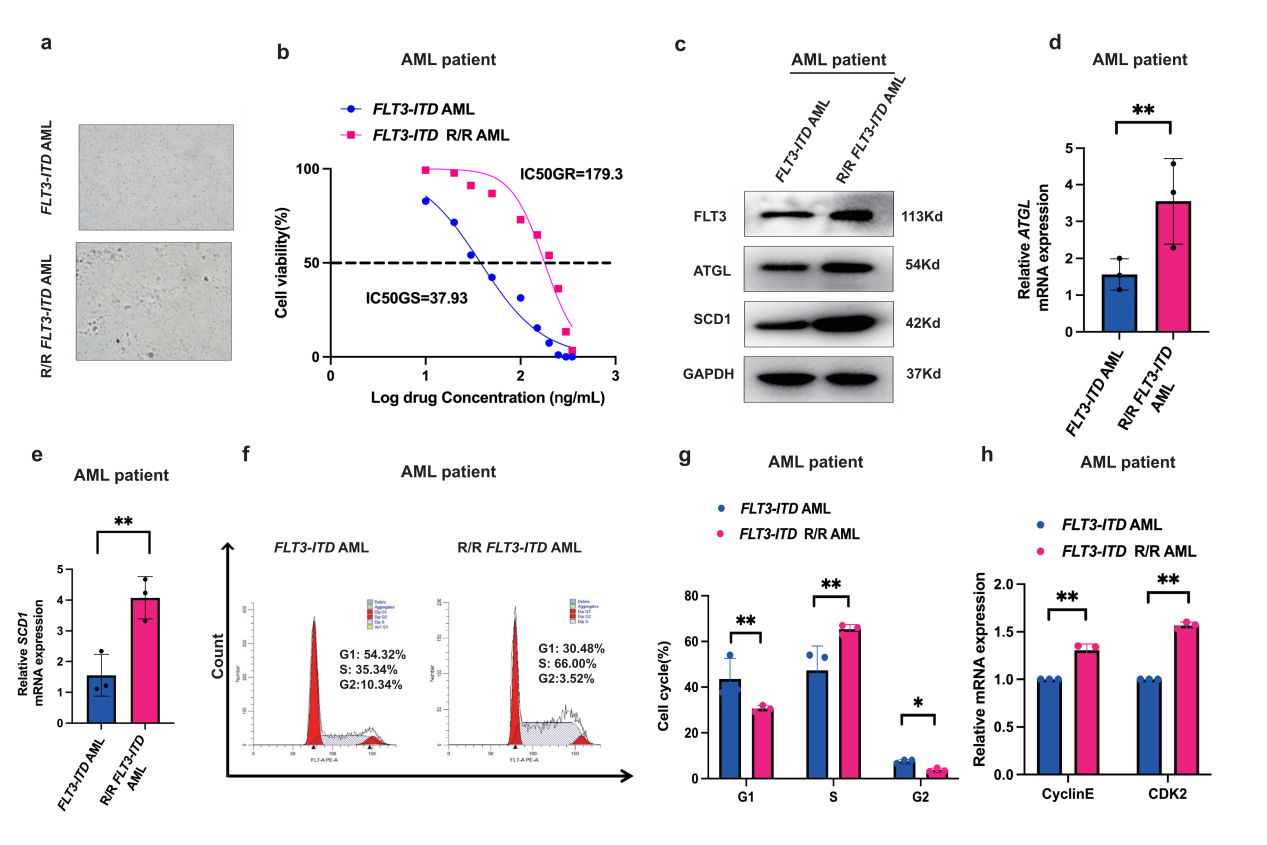 |
| --- |
| **Fig. S8 Cellular characteristics of *FLT3-*ITD AML and relapsed/refractory *FLT3-*ITD AML.** **a.** Microscopic morphology of *FLT3-*ITD mutated AML and relapsed/refractory *FLT3-*ITD mutated AML cells. **b.** Drug IC50 concentrations of gilteritinib in *FLT3-*ITD mutated AML and relapsed/refractory *FLT3-*ITD mutated AML cells. **c-e.** Expression of ATGL and SCD1 in *FLT3-*ITD mutated AML and relapsed/refractory *FLT3-*ITD mutated AML cells. **f-g.** Cell cycle changes in *FLT3-*ITD mutated AML and relapsed/refractory *FLT3-*ITD mutated AML cells. **h.** RNA expression of cell cycle genes CyclinE and CDK2 in *FLT3-*ITD mutated AML and relapsed/refractory *FLT3-*ITD mutated AML. **P* < 0.05; ***P* < 0.01; ****P* < 0.001; ns, not significant. |

| 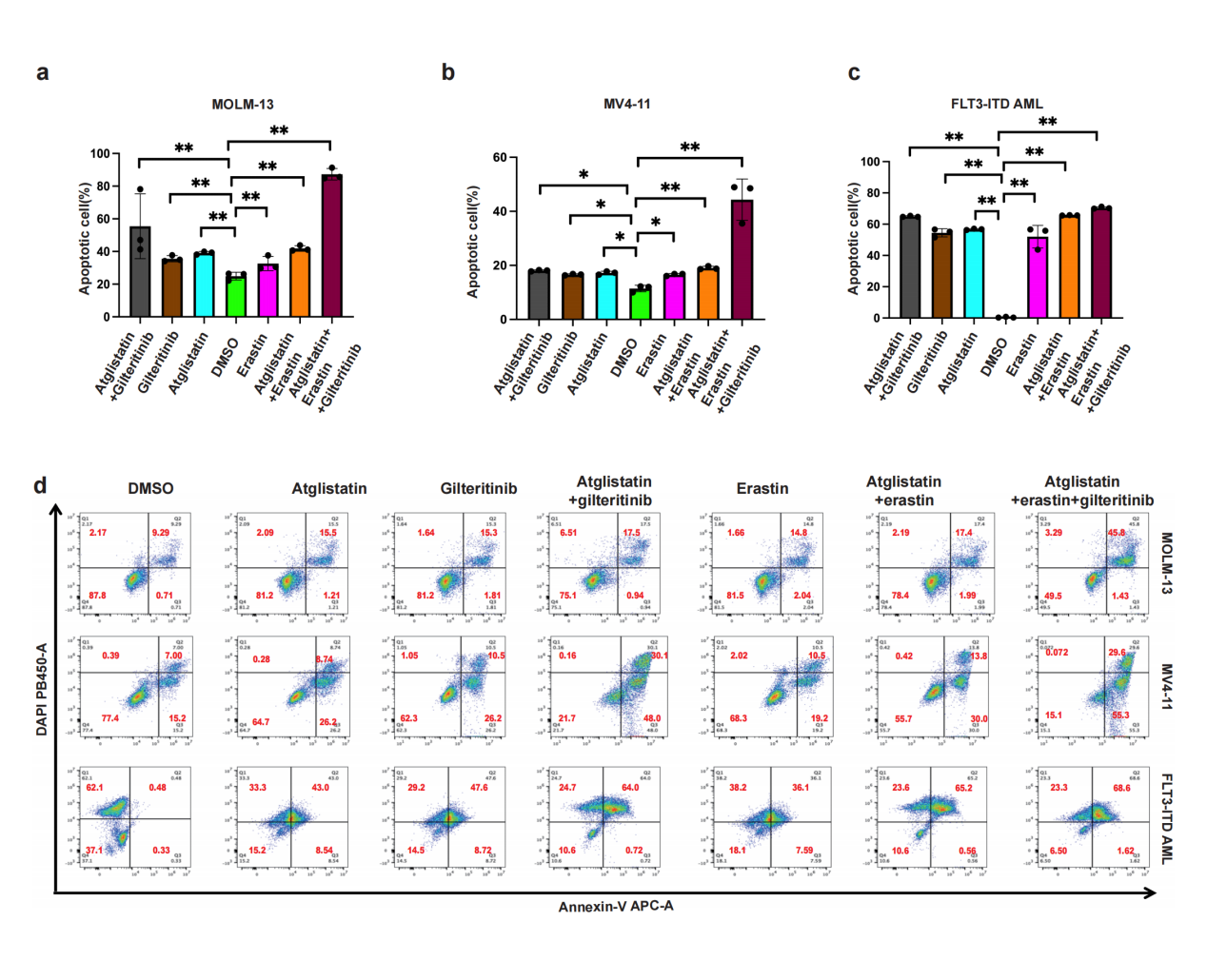 |
| --- |
| **Fig. S9 Apoptosis in MOLM-13, MV4-11, and *FLT3-*ITD mutated AML cells following treatment with the ATGL inhibitor (atglistatin, 20 μM) in combination with the FLT3 inhibitor (gilteritinib, 170 nM) or erastin (5 μM).** **P* < 0.05; ***P* < 0.01; ****P* < 0.001; ns, not significant. |

| 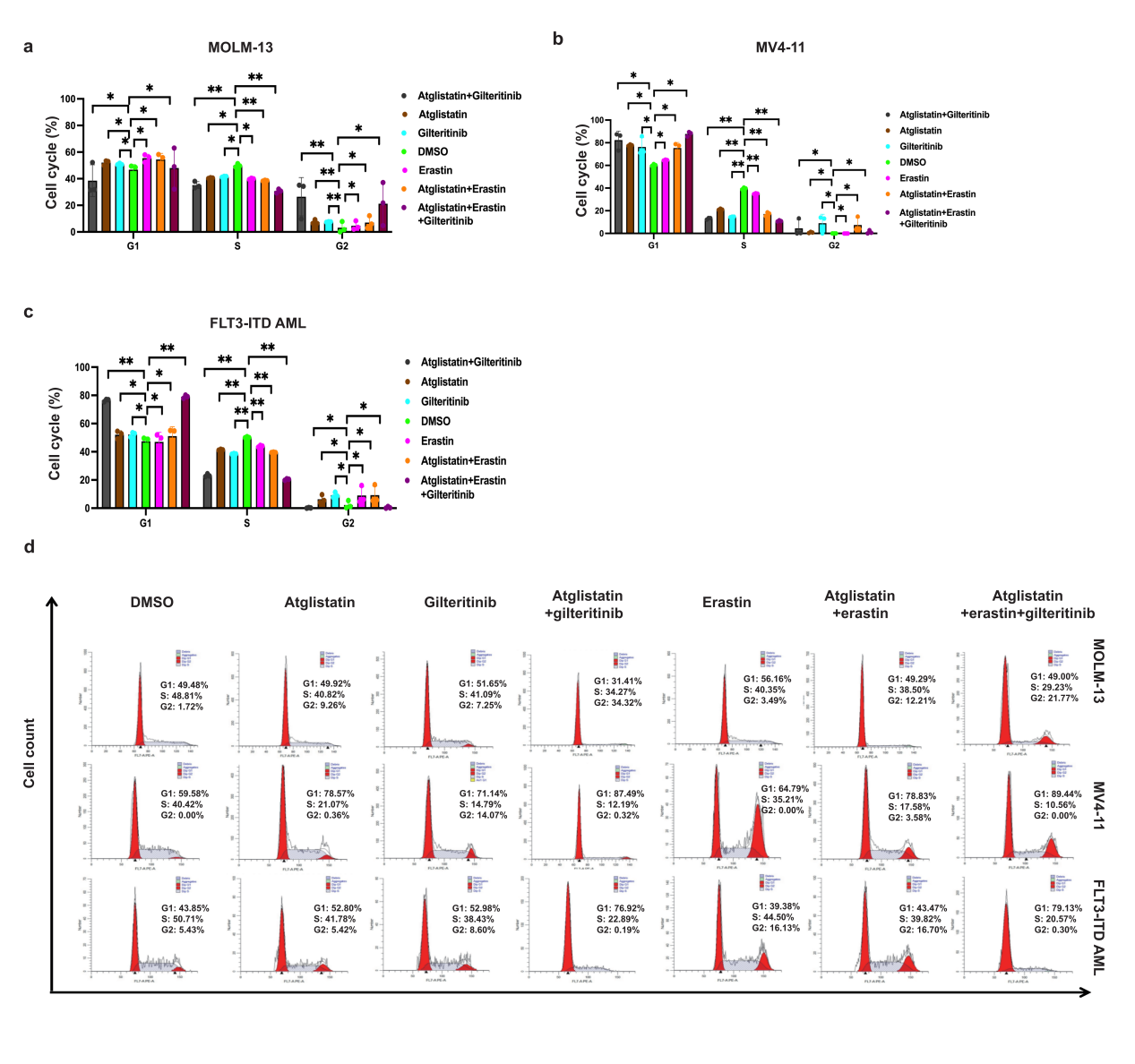 |
| --- |
| **Fig. S10 Cell cycle in MOLM-13, MV4-11, and *FLT3-*ITD AML cells following treatment with the ATGL inhibitor (atglistatin, 20 μM) in combination with the FLT3 inhibitor (gilteritinib, 170 nM) or erastin (5 μM).** **P* < 0.05; ***P* < 0.01; ****P* < 0.001; ns, not significant. |

| 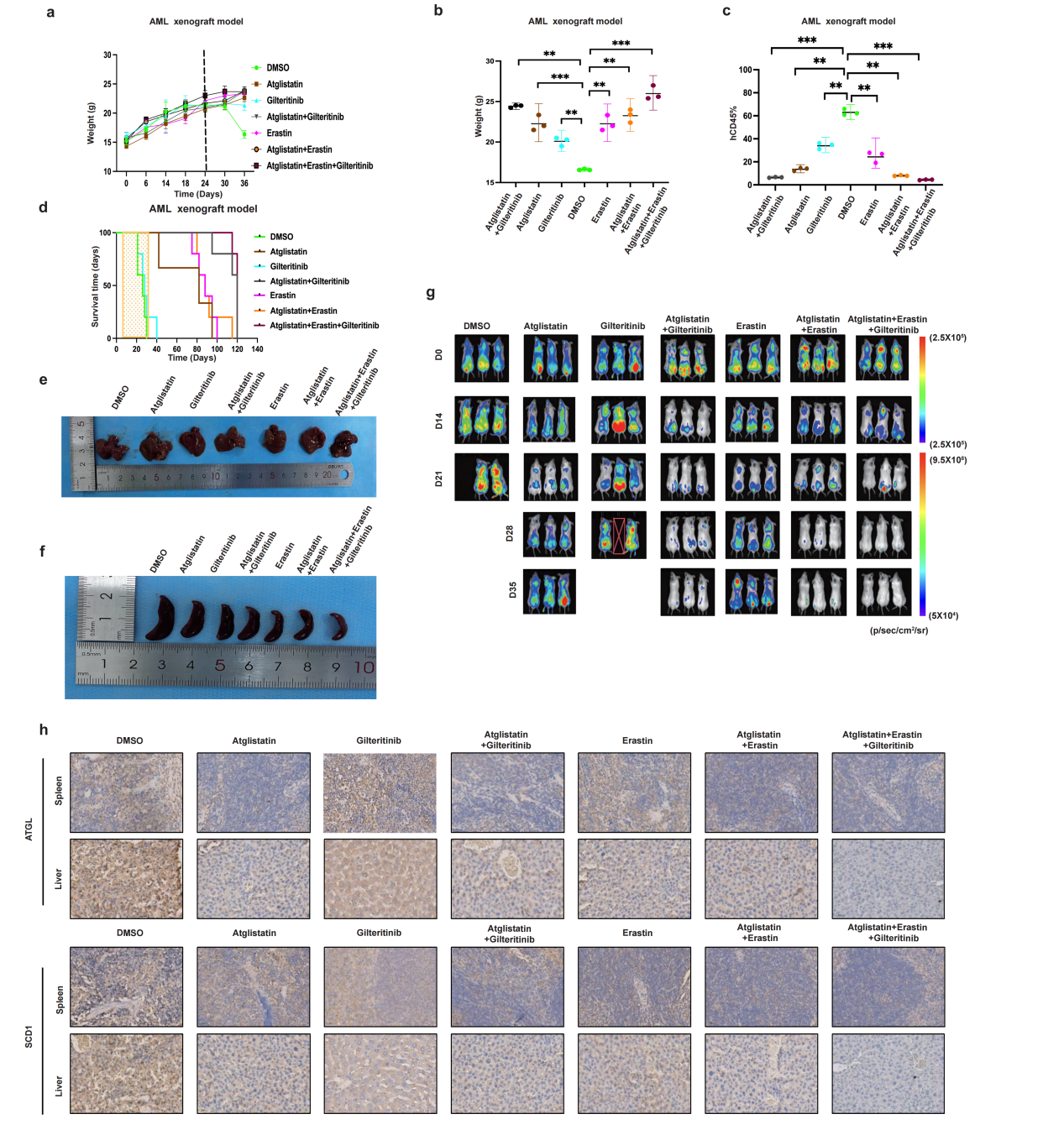 |
| --- |
| **Fig. S11 MV4-11GR (F-Luc) cells were used to construct an AML allogeneic mouse model.** **a.** Changes in mouse body weight. **b-c.** Final body weight and leukemia burden (hCD 45%) after treatment completion. **d.** Survival changes in mice observed at 120 days. **e-f.** Spleen and liver conditions of mice after treatment completion. **g.** Fluorescent in vivo imaging during treatment showing tumor changes. **h.** Representative images of immunohistochemical staining for ATGL and SCD1 in spleen and liver excised from xenograft model mice (40X), Scale bar: 25 μm. . **P* < 0.05; ***P* < 0.01; ****P* < 0.001; ns, not significant. |

**Supplemental Materials and Methods**

**RNA-seq and data analysis**

Total RNA was isolated from Sh*ATGL* and ShNC MV4-11 cells using TRIzol® reagent, and the ligated products were amplified by PCR, resulting in a final cDNA library with an average insert length of 300 ± 50 bp. finally, the final cDNA libraries were sequenced according to the supplier's recommended protocols on an illumina Novaseq™ 6000 (Hangzhou LC-Bio Science and Technology Co., Ltd., Hangzhou, China) by performing 2×150 bp paired-end sequencing (PE150). Finally, 2×150 bp paired-end sequencing (PE150) was performed on illumina Novaseq™ 6000 (LC-Bio Technology CO., Ltd., Hangzhou, China) following the protocol recommended by the vendor. Reads containing aptamer contamination, low quality bases and unrecognized bases were removed using FASTP. In addition, the quality of 10 sequences was verified using FASTP. Gene differential expression analysis was performed between two different groups (and between two samples) using DESeq2 software. Genes with a false discovery rate (FDR) of less than 0.05 and an absolute fold change of ≥2 were considered as differentially expressed genes. Differentially expressed genes were then analyzed for GO function and KEGG pathway enrichment. Whole transcriptome sequencing of peripheral blood leukocytes was performed by Shanghai Tensil Diagnostic Technology Co.

**Construction of gilteritinib-resistant strains**

Using 5 ng/ml Gilteritinib initial drug was added to MOLM-13 and MV4-11 cell lines, centrifugation and cell exchange were performed every 3 days to remove the dead cells, and continued to be added to MOLM-13 and MV4-11 cell lines in a gradient of the drug concentration, Gilteritinib concentration continued to be accumulated up to 450 ng/ml, and continued to be cultured for 6 months The Gilteritinib IC50 (IC50R) of the post-induction cell lines was measured against the Gilteritinib IC50 of the initial cell lines, and the resistance index RI = IC50R of the induced cells/initial IC50 was calculated (RI greater than 5 was considered to be the success of induction of the resistant strain), and the cell cycle of the cell lines was measured by flow cytometry for the initial and post-induction cell lines, and the microscopic morphology of the cell lines were photographed for the initial and post-induction cell lines. The initial and post-induction cell cycle was measured by flow cytometry, the microscopic morphology of the cell line was photographed, and the initial and post-induction CyclinE and CDK2 of the cell line were measured by RT- QPCR [1,2] .

**References：**

1.Li LC, Wang JD, Yang SS, Zhou Z, Zeng QF, Zheng F. [Establishment of Drug-Resistant Cell Lines of Acute Myeloid Leukemia and Correlation of Sirt1 and PGC-1α Expression Levels with Drug Resistance]. Zhongguo Shi Yan Xue Ye Xue Za Zhi. 2022 Jun;30(3):704-710.

2.Xie Y, Shangguan W, Chen Z, Zheng Z, Chen Y, Zhong Q, et al. Establishment of Sunitinib-Resistant Xenograft Model of Renal Cell Carcinoma and the Identification of Drug-Resistant Hub Genes and Pathways. Drug Des Devel Ther. 2021;15:5061-74.

**AML xenograft model**

All animal experiments were conducted in accordance with procedures approved by the Animal Ethics Committee of Chongqing Medical University (2021129), adhering to the 3Rs principle. 4–5-week-old NSG mice (10–12 g) were purchased from Vital River (Beijing, China). For in vivo experiments, mice received intravenous injections of 5 × 10^6^ ^+^ MV4-11GR (F-Luc) cells (acute myeloid leukemia). Disease implantation was assessed via bioluminescence imaging (BLI) on day 7, with photon emission from MV4-11GR (F-Luc) cells quantified using Live Image software

after the injection of 200 mL of D-luciferina. Drug injections were administered according to the experimental protocol on day 7, initiating in vivo fluorescence monitoring from day 1. Subsequent assessments occurred every 7 days until the 28-day treatment endpoint. All other evaluation methods followed the procedures detailed in the animal experimentation section of the manuscript.
