# Supplementary material for: ATGL suppresses ferroptosis in acute myeloid leukemia cells by modulating the CEBPα/SCD1 axis and induces gilteritinib resistance
Source: Cell Death Dis. 2026 Jan 9;17(1):171. doi: 10.1038/s41419-025-08388-0 (PMC12876964; doi:10.1038/s41419-025-08388-0)

Figure1 I

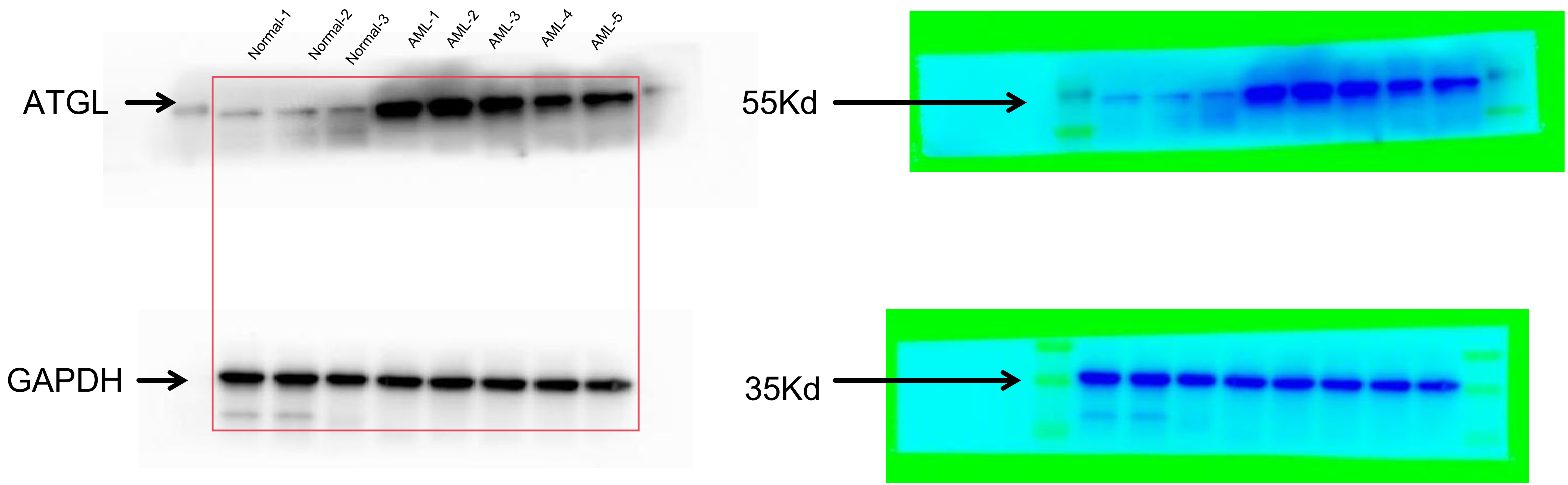
Normal-1
Normal-2
Normal-3
AML-1
AML-2
AML-3
AML-4
AML-5
ATGL
GAPDH
55Kd
35Kd

Figure2 a

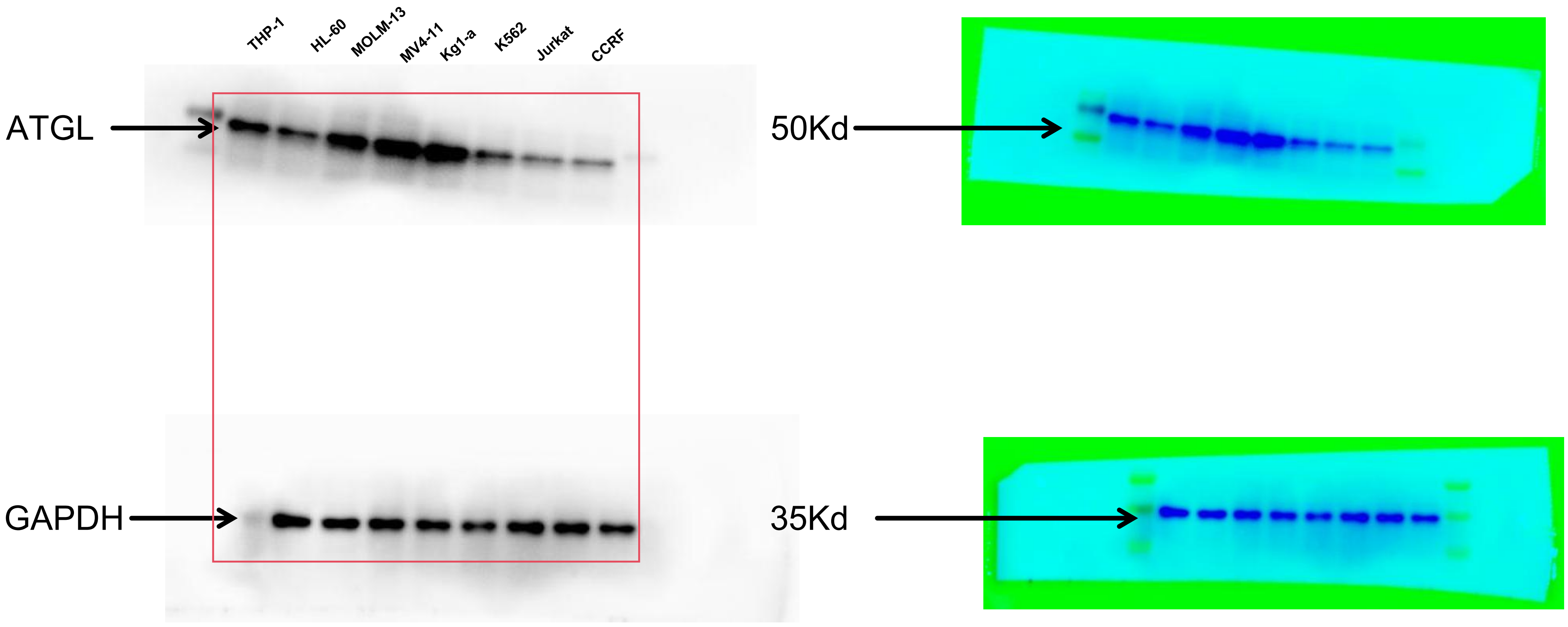
THP-1
HL-60
MOLM-13
MV4-11
Kg1-a
K562
Jurkat
CCRF
ATGL
GAPDH
50Kd
35Kd

Figure2 b

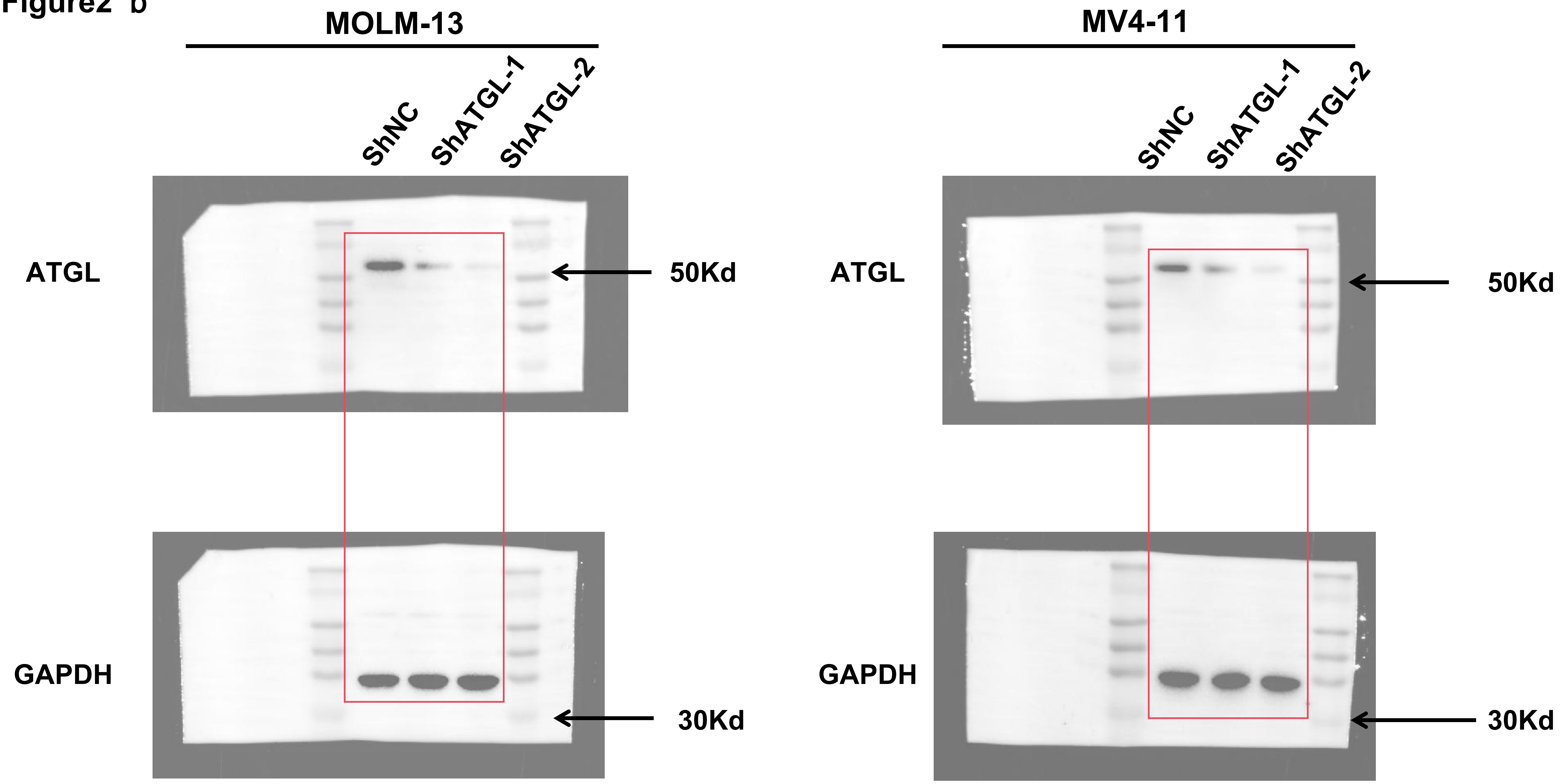
MOLM-13
ShNC
ShATGL-1
ShATGL-2
ATGL
50Kd
GAPDH
30Kd
MV4-11
ShNC
ShATGL-1
ShATGL-2
ATGL
50Kd
GAPDH
30Kd

Figure 2g

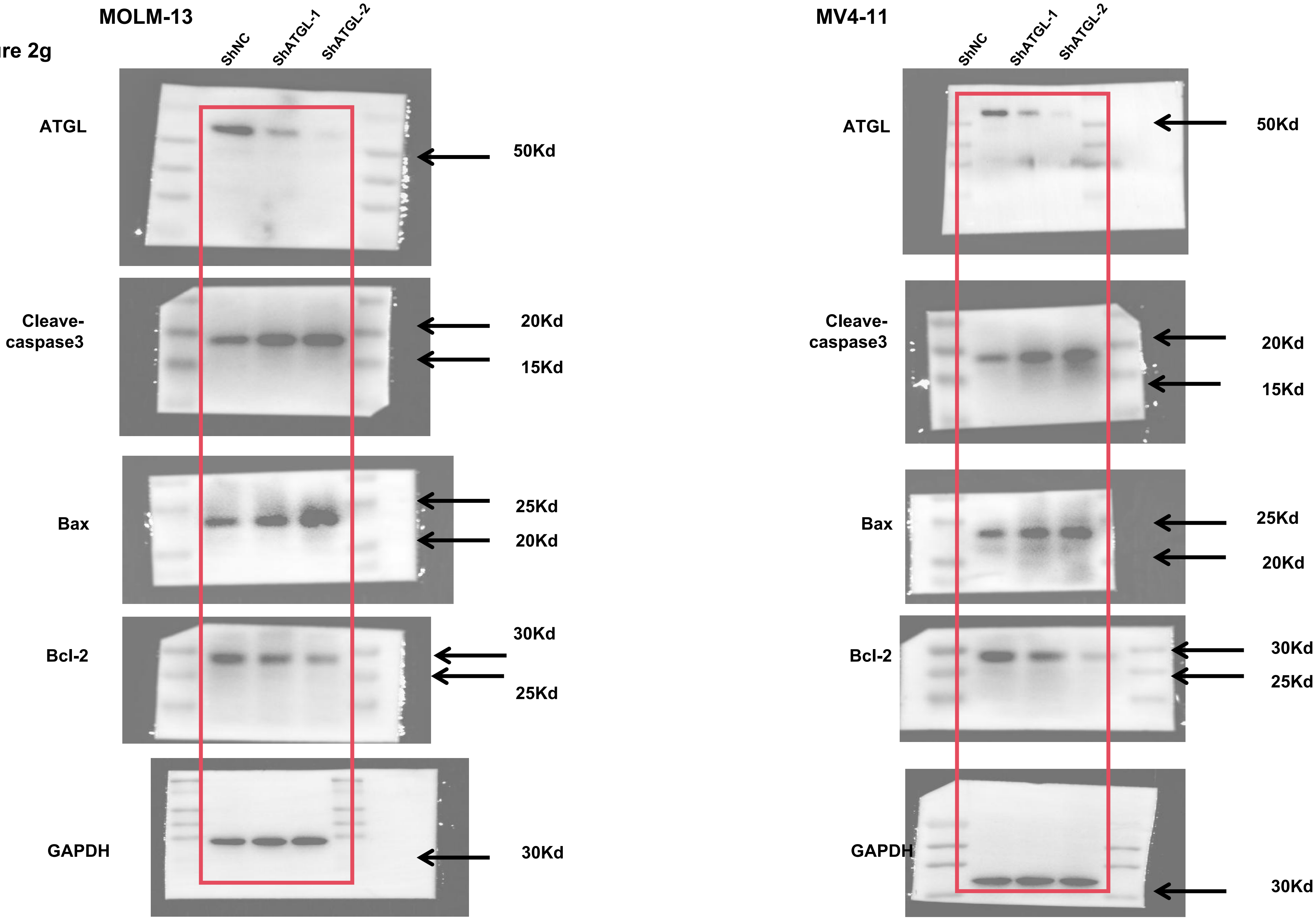
MOLM-13
ShNC
ShATGL-1
ShATGL-2
ATGL
50Kd
Cleave-caspase3
20Kd
15Kd
Bax
25Kd
20Kd
Bcl-2
30Kd
25Kd
GAPDH
30Kd
MV4-11
ShNC
ShATGL-1
ShATGL-2
ATGL
50Kd
Cleave-caspase3
20Kd
15Kd
Bax
25Kd
20Kd
Bcl-2
30Kd
25Kd
GAPDH
30Kd

Figure 3k

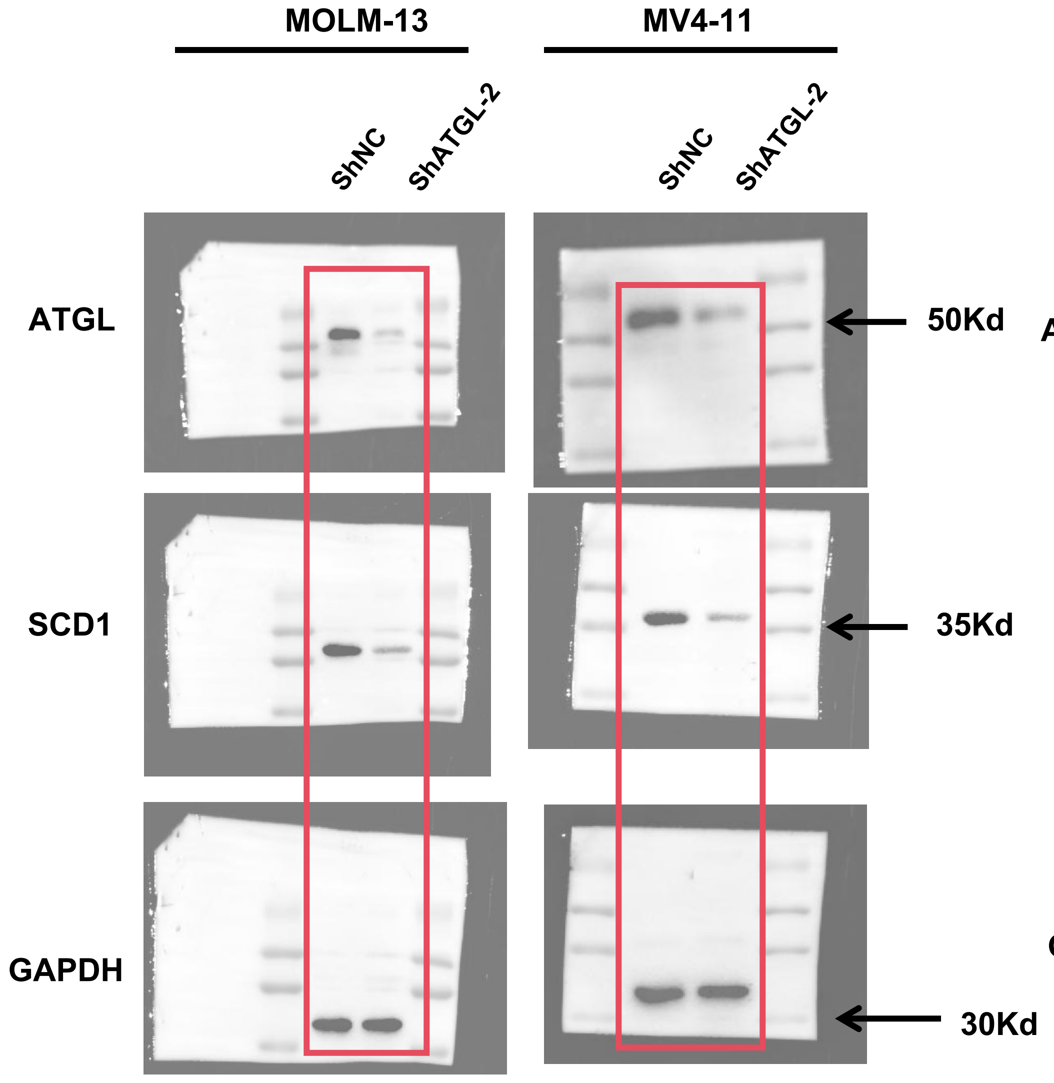

Figure 3n

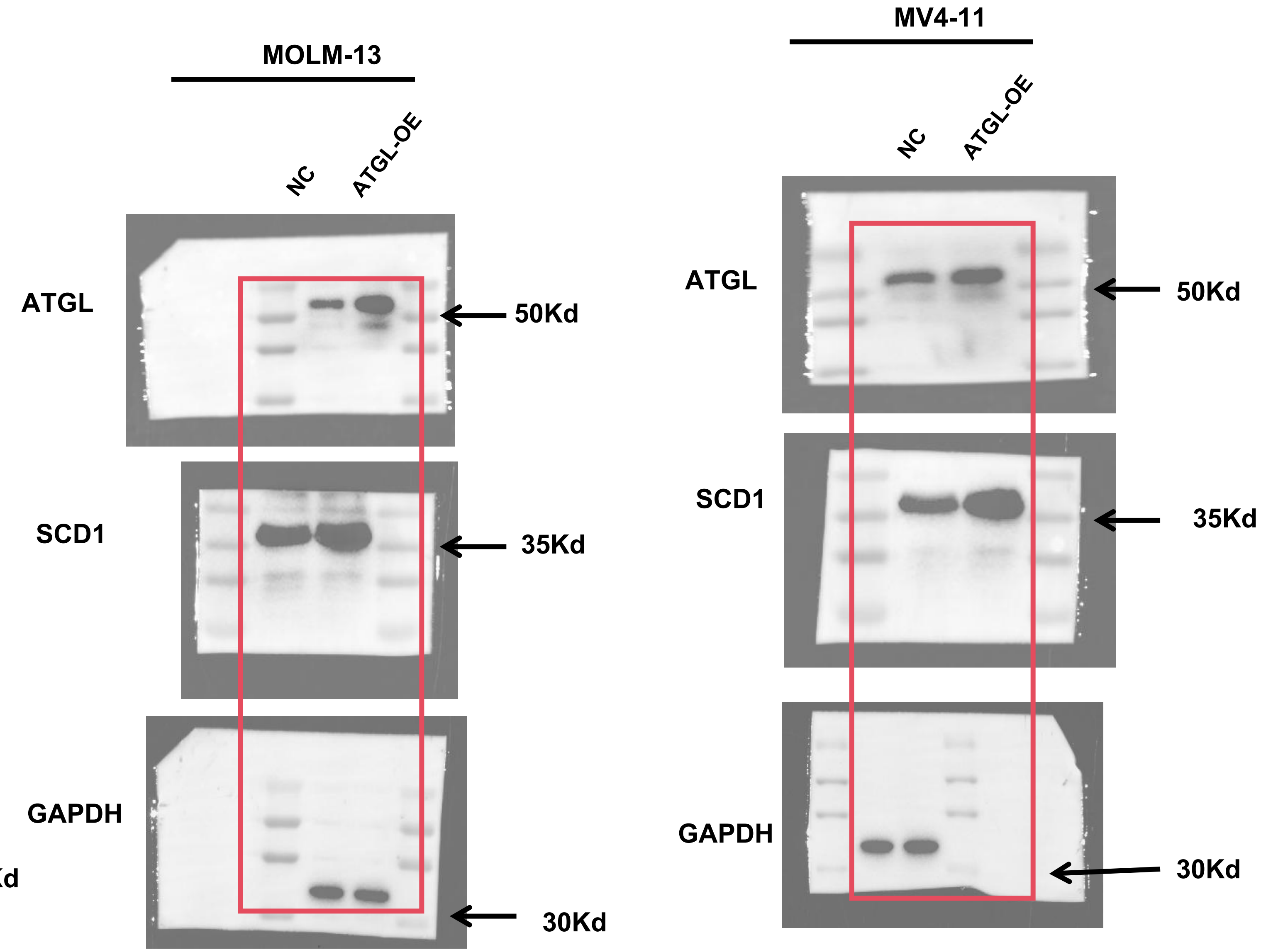

Fig. 4c

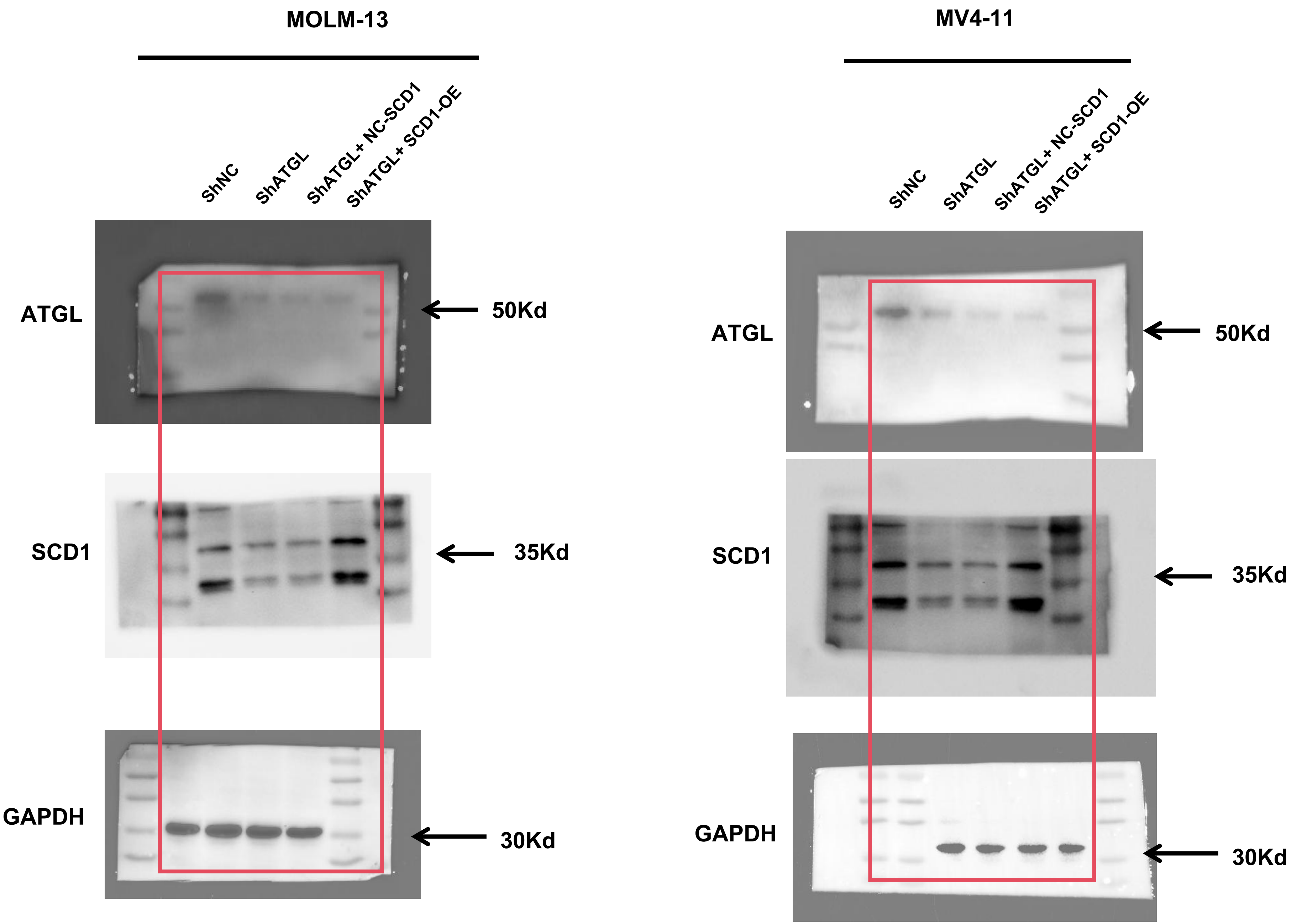
MOLM-13
ShNC
ShATGL
ShATGL+ NC-SCD1
ShATGL+ SCD1-OE
ATGL
50Kd
SCD1
35Kd
GAPDH
30Kd
MV4-11
ShNC
ShATGL
ShATGL+ NC-SCD1
ShATGL+ SCD1-OE
ATGL
50Kd
SCD1
35Kd
GAPDH
30Kd

Figure. 4k

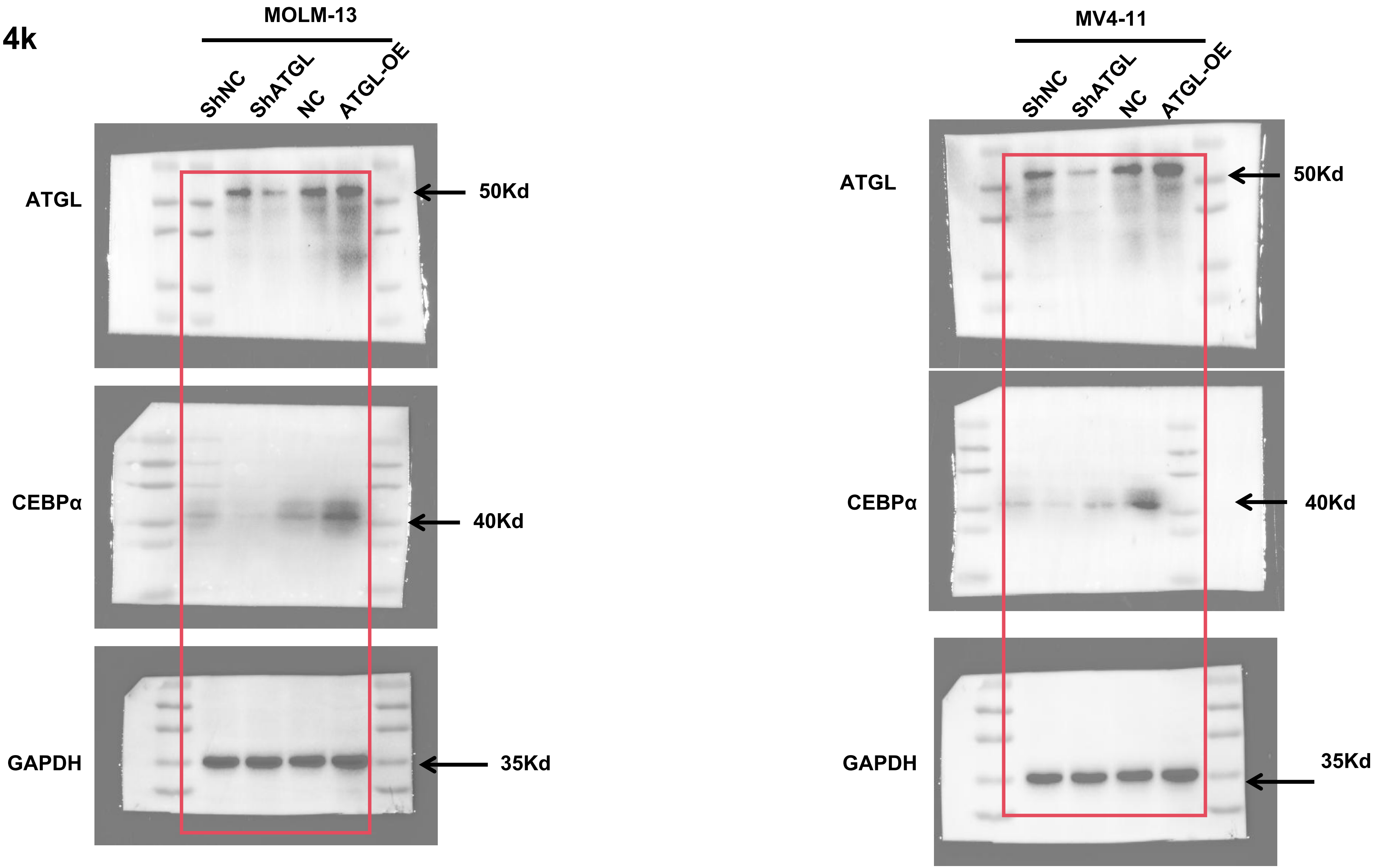
MOLM-13
ShNC
ShATGL
NC
ATGL-OE
ATGL
50Kd
CEBPα
40Kd
GAPDH
35Kd
MV4-11
ShNC
ShATGL
NC
ATGL-OE
ATGL
50Kd
CEBPα
40Kd
GAPDH
35Kd

Figure.4o

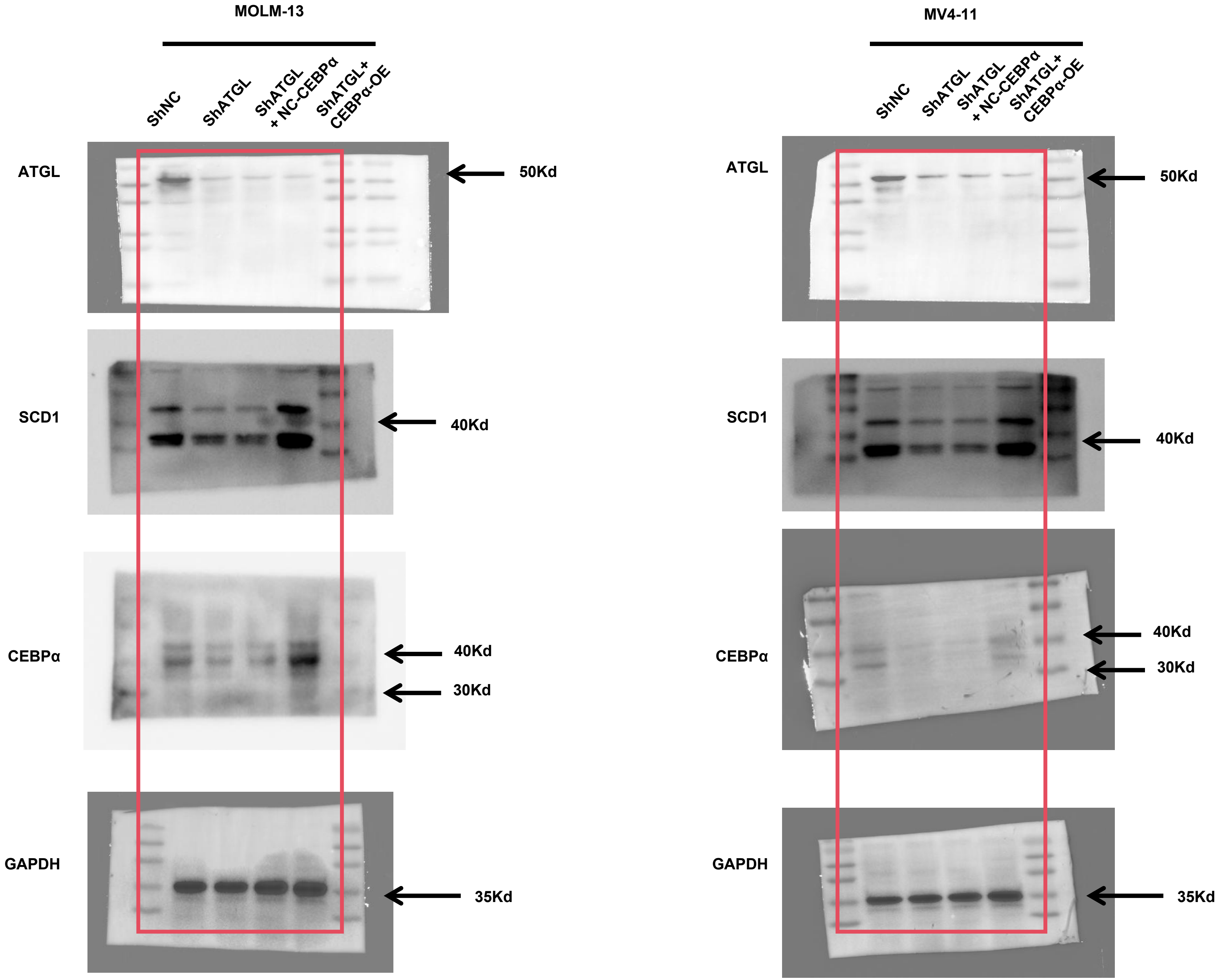

MOLM-13
MV4-11
ShNC
ShATGL
ShATGL + NC-CEBPα
ShATGL+ CEBPα-OE
ATGL
SCD1
CEBPα
GAPDH
50Kd
40Kd
30Kd
35Kd

Figure. 6b

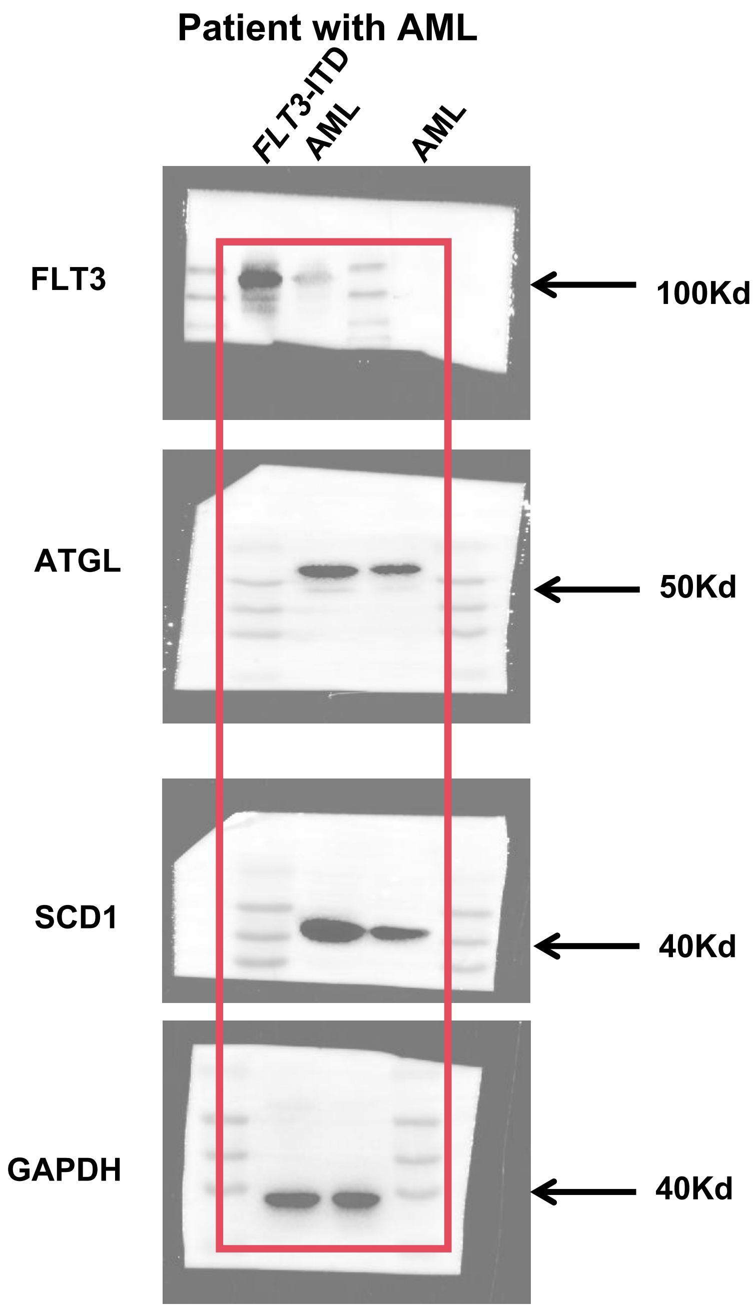
Patient with AML
FLT3-ITD AML
AML
FLT3
ATGL
SCD1
GAPDH
100Kd
50Kd
40Kd
40Kd

Figure. S1a

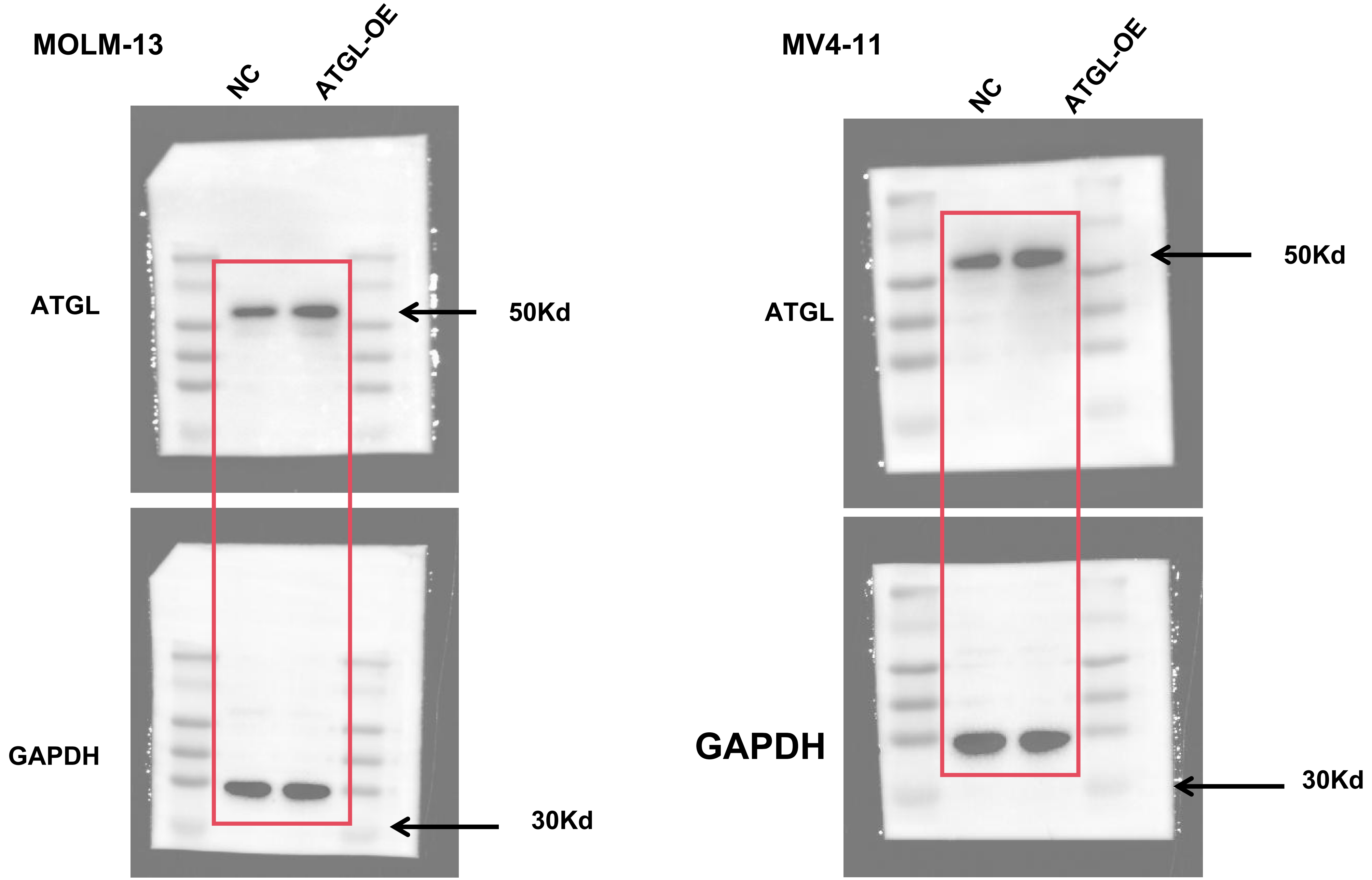
MOLM-13
NC
ATGL-OE
ATGL
50Kd
GAPDH
30Kd
MV4-11
NC
ATGL-OE
ATGL
50Kd
GAPDH
30Kd

Figure. S1g

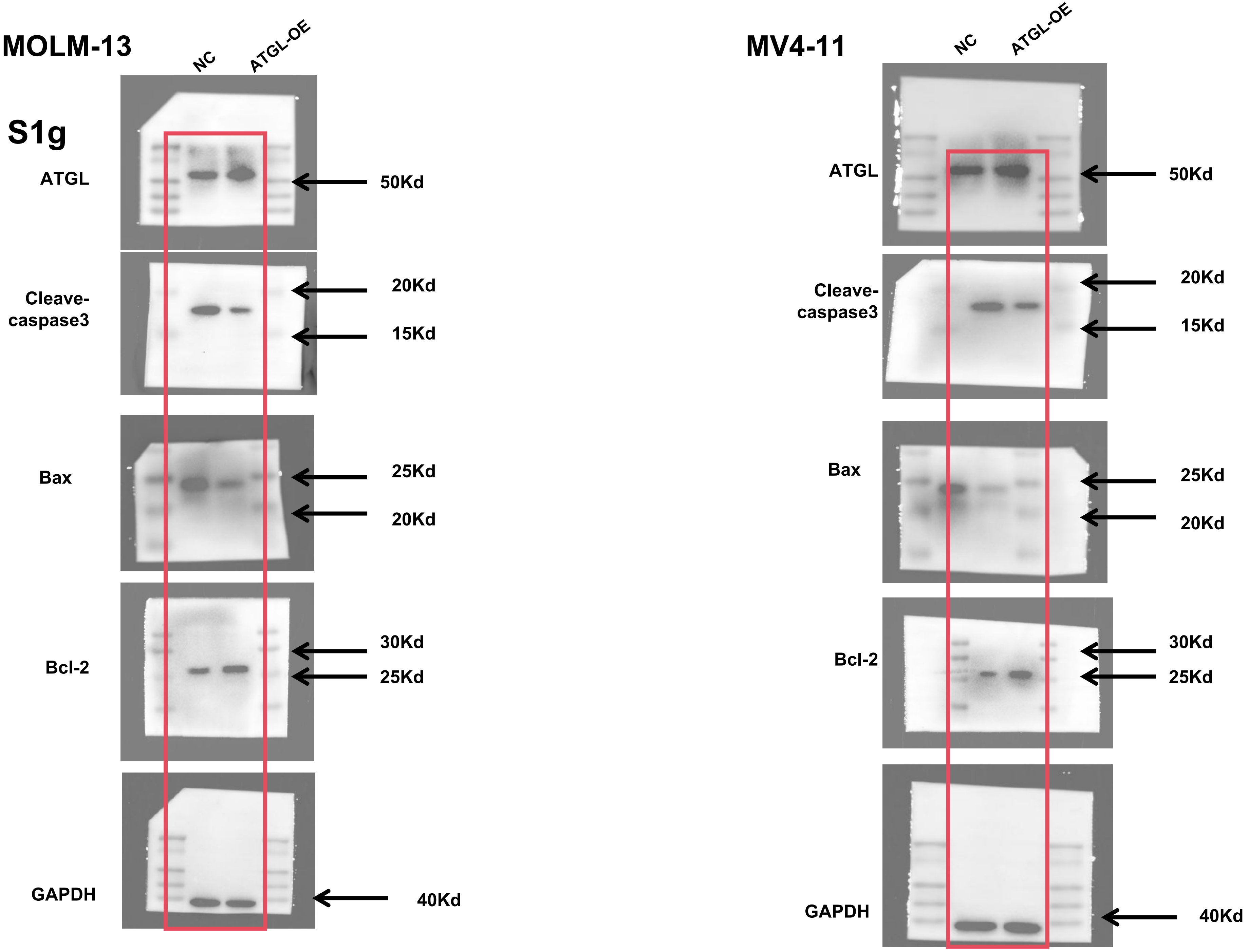
MOLM-13
NC
ATGL-OE
ATGL
50Kd
Cleave-
caspase3
20Kd
15Kd
Bax
25Kd
20Kd
Bcl-2
30Kd
25Kd
GAPDH
40Kd
MV4-11
NC
ATGL-OE
ATGL
50Kd
Cleave-
caspase3
20Kd
15Kd
Bax
25Kd
20Kd
Bcl-2
30Kd
25Kd
GAPDH
40Kd

Figure. S3a

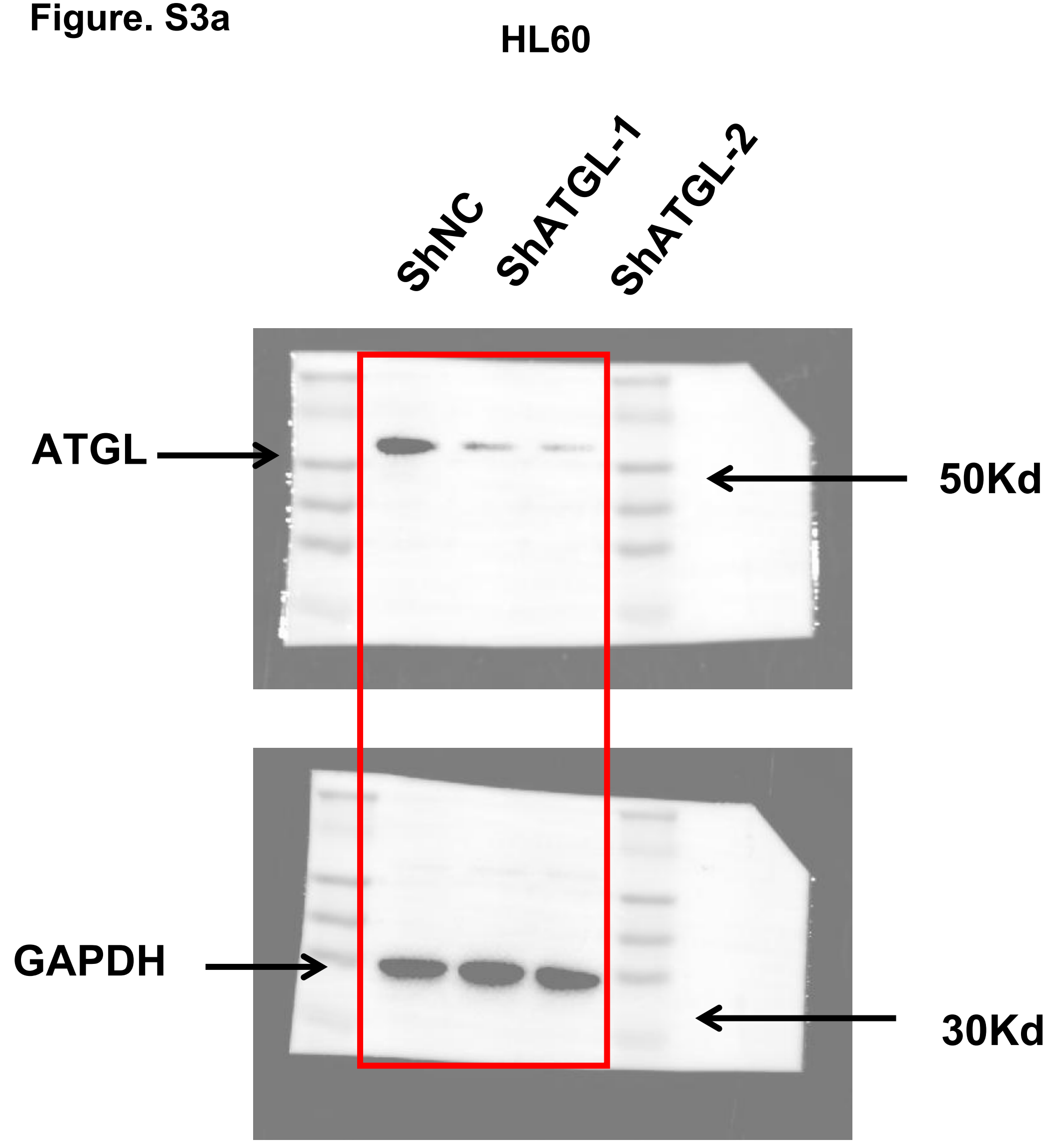

Figure. S4a

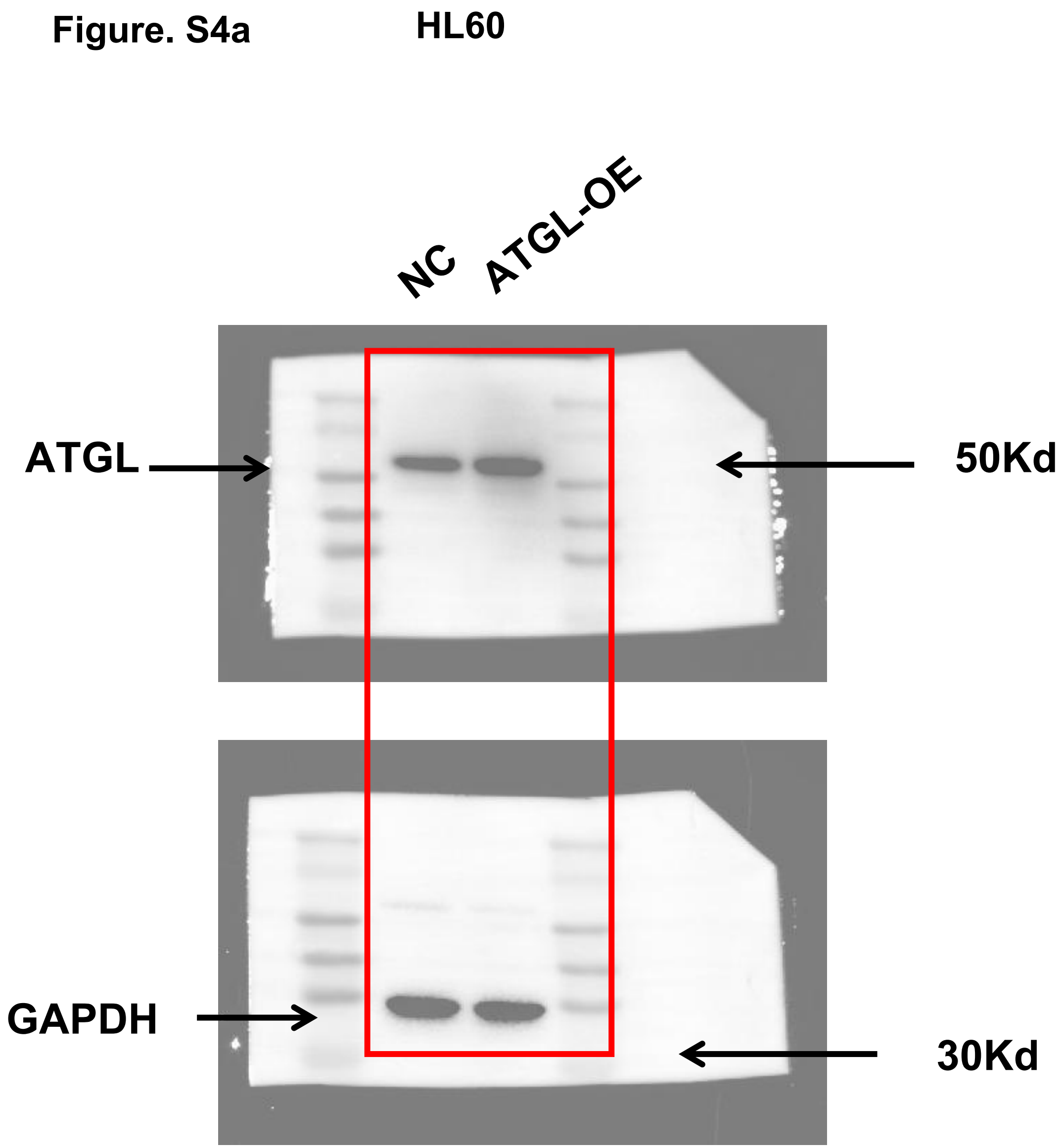

Figure. S3e

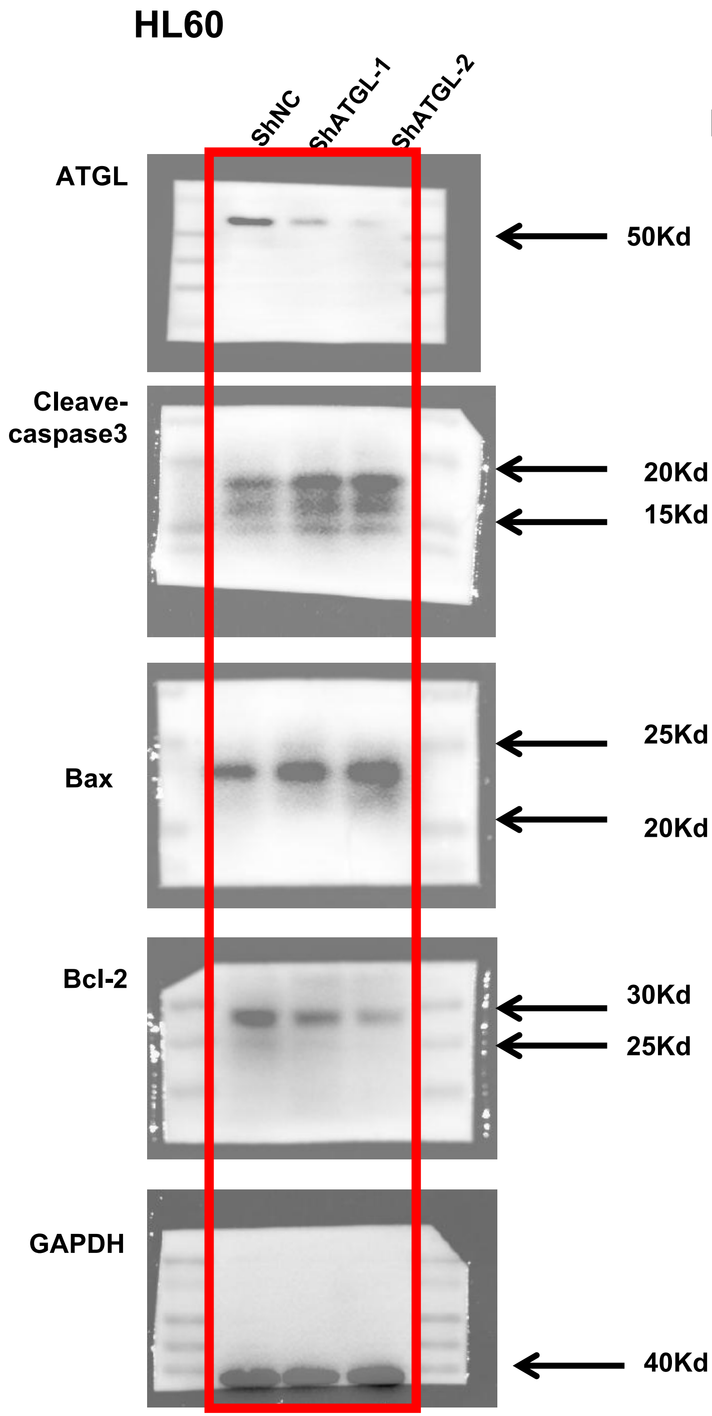

Figure. S4e

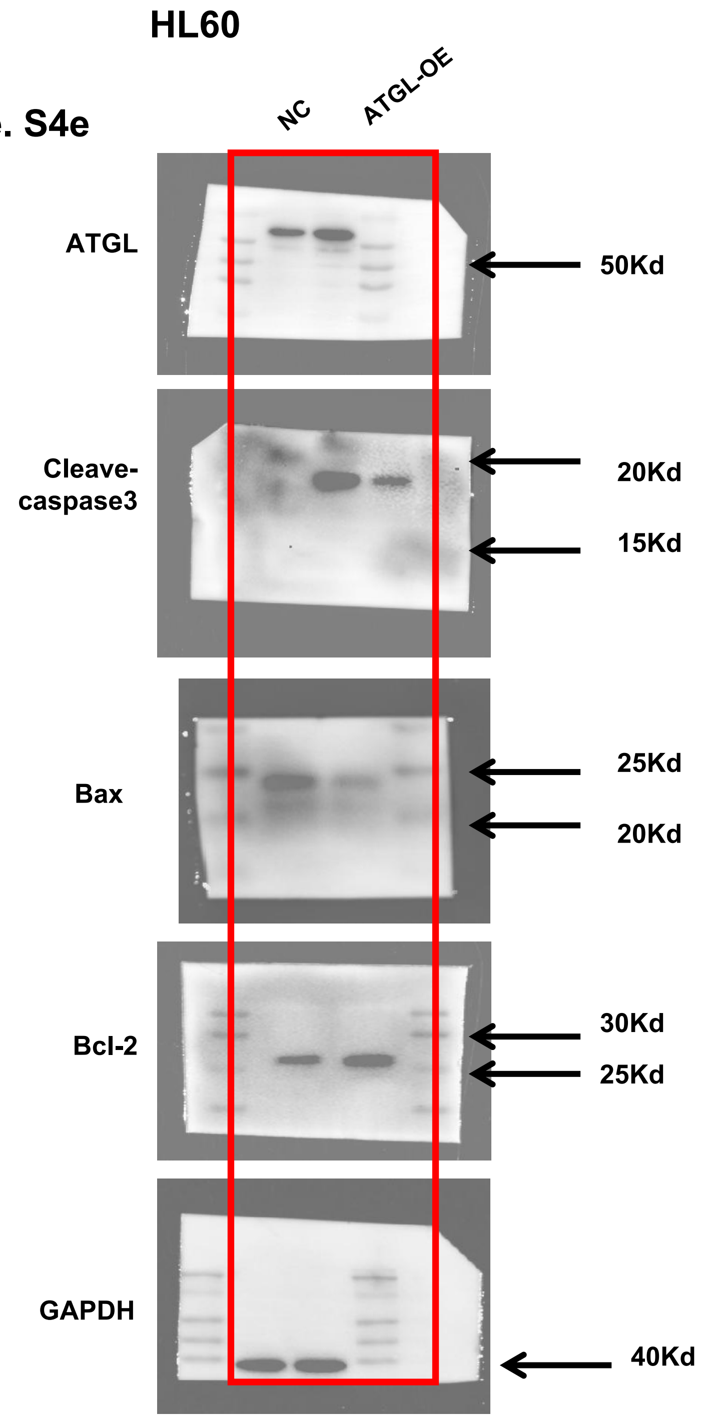

Figure. S7g

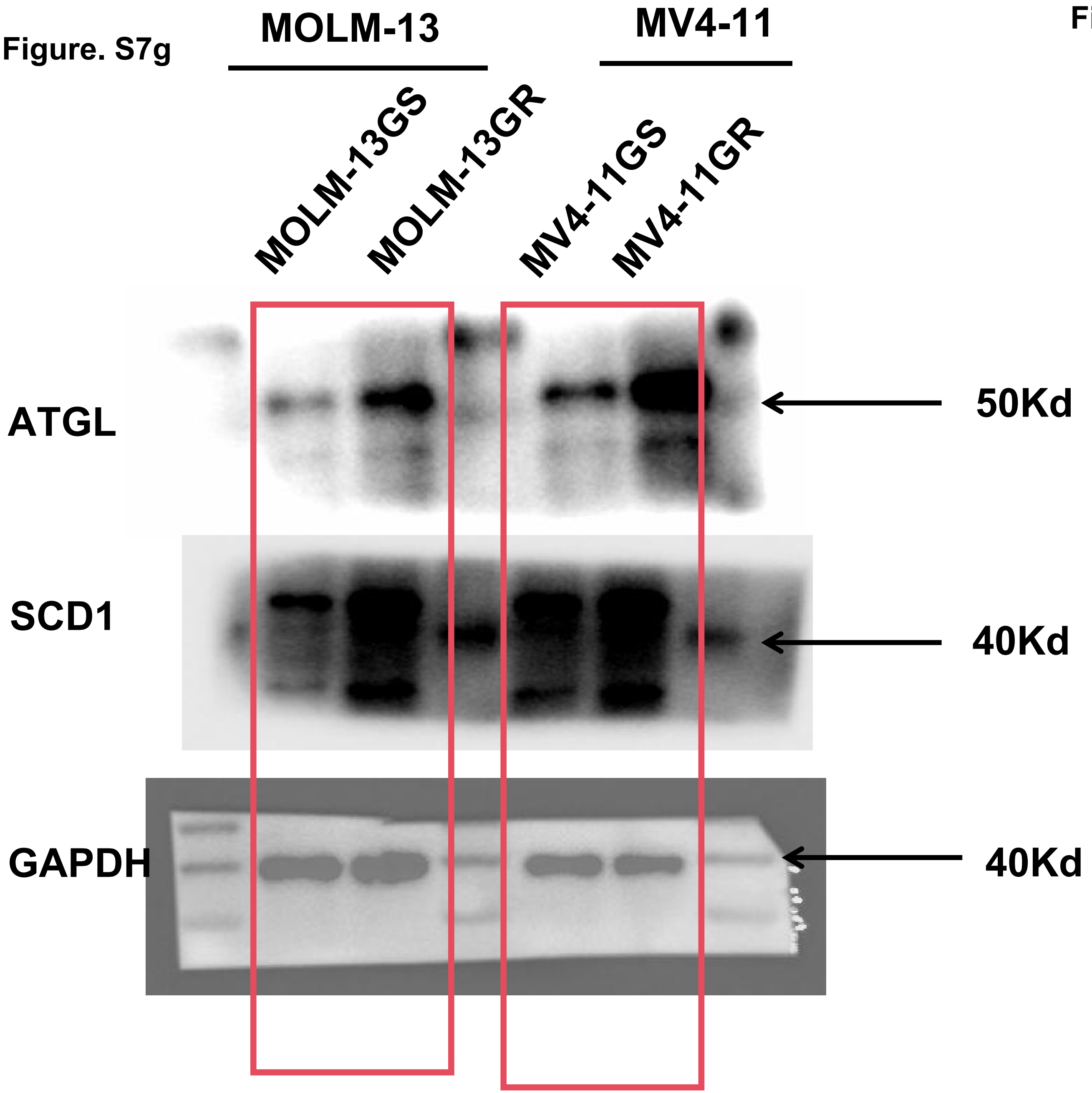

Figure. S8c

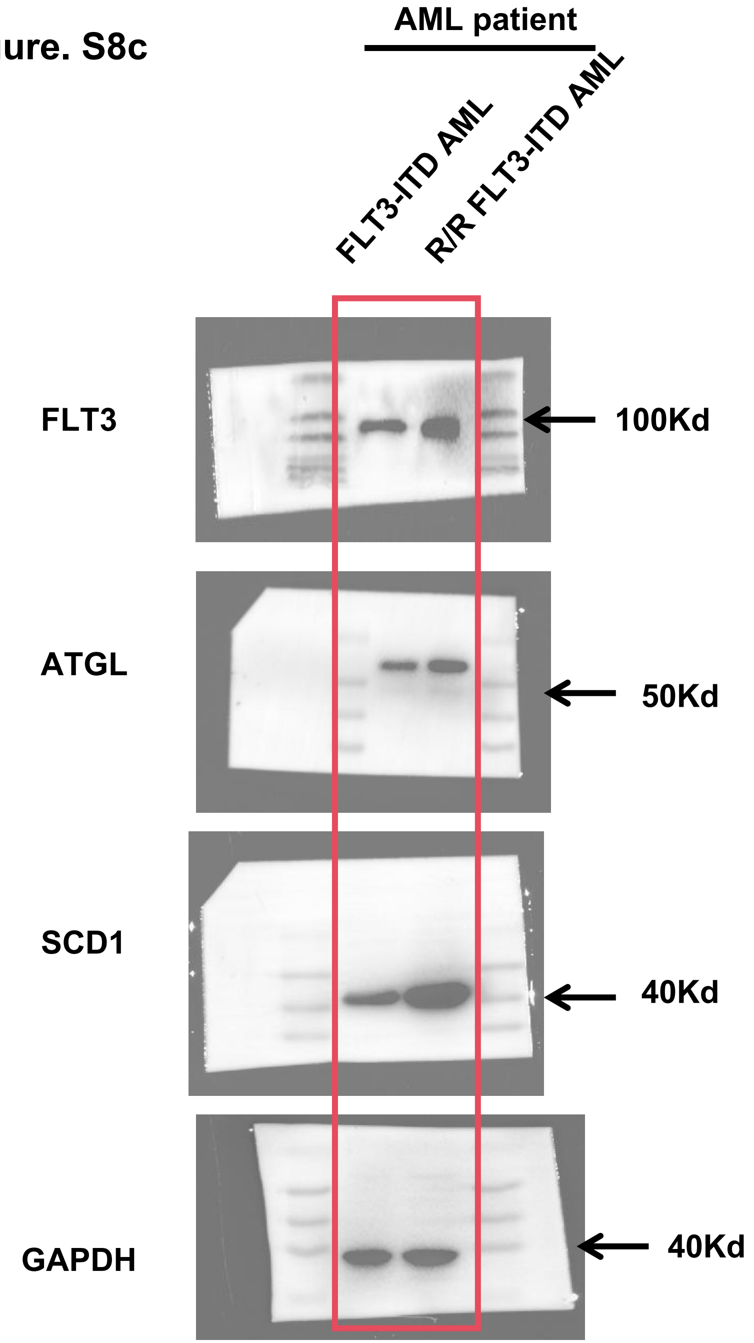

Supplement: Supplementary file 2 — Original Data [file 41419_2025_8388_MOESM2_ESM.pdf]
